# A systematic review of maternal antidepressant use in pregnancy and short- and long-term offspring’s outcomes

**DOI:** 10.1007/s00737-017-0780-3

**Published:** 2017-10-12

**Authors:** Stephanie L. Prady, Inna Hanlon, Lorna K. Fraser, Antonina Mikocka-Walus

**Affiliations:** 1 Department of Health Sciences, University of York, Seebohm Rowntree Building, York, YO10 5DD UK; 2 School of Psychology, Deakin University, Burwood, VIC Australia

**Keywords:** Antidepressants, Low birth weight, Neurodevelopment, Neurobehaviour, Pregnancy

## Abstract

The relative safety of antidepressants during pregnancy has received substantial attention, but most syntheses fail to account for mental illness effects. We aimed to evaluate the literature comparing low birth weight (LBW) and neurodevelopmental and neurobehavioural outcomes for children whose mothers took antidepressants in pregnancy compared to those whose mothers had common mental disorders, or symptoms, but who did not take antidepressants during pregnancy. A systematic review was conducted searching PubMed, MEDLINE, PsycINFO and Embase in January 2015. A modified version of the Newcastle Ottawa Scale was used to assess study quality. Eleven cohort studies were included: four reporting a LBW outcome (all with higher risk of bias) and seven reporting a neurodevelopmental outcome (five with higher risk of bias). We found only limited evidence of gestational age-adjusted LBW in exposed children in two studies which had a higher risk of bias and did not control for depressive symptom severity. Only five (7.5%) neurodevelopmental outcomes and one (12.5%) neurobehavioural outcome showed evidence of a statistically significant effect, three out of four were from studies with a higher risk of bias. There is little robust evidence indicating a detrimental effect of antidepressant use during pregnancy on LBW and neurodevelopmental and neurobehavioural outcomes. More rigorous study designs are needed.

**Electronic supplementary material:**

The online version of this article (10.1007/s00737-017-0780-3) contains supplementary material, which is available to authorized users.

## Introduction

Depression and anxiety commonly occur in pregnancy, and there exist a range of effective treatments (NICE 2015). Psychological treatments are preferred for mild to moderate uncomplicated episodes during pregnancy; however, more severe or recurrent episodes are indications for pharmacological treatment (Buist et al. 2005). Having an effective treatment in place during pregnancy is important, as, in addition to the distress and suffering they cause, common mental disorders have been associated with increased risks of preterm delivery, low birth weight (LBW) and neurodevelopmental or neurobehavioural problems or delays in the offspring, such as cognitive, emotional and behavioural development (Agnafors et al. 2013; Grote et al. 2010; O’Connor et al. 2002).

The relative safety of antidepressant treatment during pregnancy has received substantial research attention. However, among the numerous examples of previous systematic reviews on the subject (Bromley et al. 2012; Fenger-Gron et al. 2011; Gentile and Galbally 2011; Lattimore et al. 2005; McDonagh et al. 2014; Previti et al. 2014; Ross et al. 2013; Simoncelli et al. 2010; Udechuku et al. 2010), only one (Ross et al. 2013) sought to assess the effects of antidepressant exposure against being depressed but unexposed to antidepressants. This indicates that the vast majority of research, and syntheses, has compared effects of exposure against asymptomatic and unexposed women. Estimating effects compared to healthy unexposed women will tend to over-estimate the risks of exposure relative to the actual clinical problem, which is ‘Is it less harmful to the child [and the mother] to continue with antidepressants, or remain medically untreated during pregnancy?’ Accurate estimates from high-quality research are necessary to ensure that clinical decisions are properly informed.

### Aims of the study

We sought to systematically evaluate the literature comparing outcomes for children of women who took antidepressants compared to those whose mothers had common mental disorders, or symptoms, during pregnancy. We selected two groups of outcomes: (1) LBW to provide current evidence given a previous review (Ross et al. 2013) is now outdated and (2) neurodevelopmental and neurobehavioural outcomes, for which the evidence base is more sparse and no synthesis has focused on reporting effects compared to a non-healthy control group. Our aims were twofold: (1) to report outcomes in these two areas and (2) to examine in detail study methods and potential areas of bias.

## Materials and methods

Ethical approval was not sought as we reviewed previously published studies.

### Selection of studies

See Box 1 for the summary of the inclusion criteria. Included studies were limited to articles published in peer-reviewed journals and to papers published in English.

Studies were excluded if they:

- Reported a citation for which a full text was not available or was not available in English
- Were abstracts
- Did not have a comparison group or lacked the outcomes of interest
- Were conducted with non-human subjects
- Were meta-analyses, systematic reviews, literature reviews or practice guidelines as the review was concerned with original research (reference lists of relevant systematic reviews were searched for potentially relevant studies)
- Were case reports/case series as their samples are typically small and the potential for bias is high
- Were cross-sectional
- Reported insufficiently defined assessments of neurodevelopmental outcomes (e.g. the timing of outcome assessment, measurement tools or units of measurement were not reported)

Box 1: Inclusion criteria

**Table:** 

Study design	Randomised controlled trials and prospective (prospective cohort) or retrospective (case-controlled studies, retrospective cohort) observational studies
Population(s)	Children whose mothers who took antidepressants while pregnant
Exposure(s)	Antidepressants
Comparators	Children whose mothers were depressed or anxious and non-exposed to antidepressants (not treated or undergoing psychological, or alternative treatments such as light therapy, massage therapy, exercise or omega-3 fatty acid supplementation).
Outcomes	At least one of the following outcomes: LBW of infant/neonate is birth weight < 2.500 kg or small for gestational age (SGA), defined as weight for gestation < 10th (or 5th) percentile or birth weight is lower than 2 standard deviations below the mean value for the gestational age. Neurodevelopmental outcomes: emotional, behavioural, IQ, speech and language, motor development, attention and other forms of cognitive functioning and neurodevelopmental diagnoses (autistic spectrum disorder, attention deficit hyperactivity disorder and pervasive developmental disorder) of infants and young child that are measured at least 4 weeks after birth, using rating scales carried out by trained staff.

### Data sources

To identify all available studies meeting the inclusion criteria, a computerised search was performed in PubMed, MEDLINE, PsycINFO and Embase in January 2015 without limits on year, language or study design. The search was conducted by one author (IH) with support from an academic liaison librarian. The Cochrane library and other databases were searched to identify any relevant systematic reviews. A manual search was also conducted on bibliographies of these systematic reviews and others identified through the main electronic search, and reference lists of included articles.

### Search strategy

An example search strategy can be found in Appendix 1.

### Screening and study selection

Screening was conducted by one author (IH). Titles and abstracts were screened and the majority excluded based on irrelevance to the search criteria, duplication or being published in languages other than English. Full texts of potentially relevant studies were then obtained and screened against the inclusion and exclusion criteria. The reference lists of relevant systematic reviews and included studies were hand searched.

### Data extraction

Data extraction of clinically and methodologically relevant information was performed by a single author (SLP) and checked by a second (A M-W). Where data were indicated to be reported in a linked paper, we also extracted data from that publication. The following data were extracted: first author; year of publication; study design; location; recruitment method and when recruited, number recruited (in each category for exposure and control group); reported characteristics; antidepressants studied (including definition, ascertainment and prevalent use); maternal mental disorder (including definition, ascertainment and prevalence); outcomes (including definition, ascertainment and prevalence); other treatments; and results (including numbers analysed). For the neurodevelopmental outcomes, we extracted outcome data at the last time point in the study.

### Quality appraisal

We modified the Newcastle Ottawa Scale (NOS) to assess the quality of included studies (Reeves et al. 2008). The NOS has eight items split into three dimensions: selection, comparability and outcome/exposure that is dependent on the study type—outcome (cohort studies)/exposure (case-control studies). A point rating is used with one point maximum for each item except for the comparability section, which allows a two-point allocation for factors deemed important to the review question. For the comparability section of low birth weight studies, we allocated one point if the study had adjusted for depression/anxiety severity during pregnancy, and one point if it had adjusted for at least two of the following factors: (1) other psychoactive drug use during pregnancy, (2) smoking during pregnancy and (3) drinking during pregnancy. For the comparability section of the neurodevelopmental and neurobehavioural outcome studies, we allocated half a point if the study had adjusted/otherwise controlled for depression/anxiety severity during pregnancy, half a point if they had adjusted for depression severity measured at any point after delivery, half a point if they had adjusted for socio-economic status or position (measured in income, education, area deprivation, individual deprivation, home-ownership, etc. either pre- or post-natally) and half a point if they had adjusted/controlled for at least two of the following factors: (1) other psychoactive drug use during pregnancy, (2) smoking in pregnancy, (3) drinking during pregnancy, (4) intrauterine growth restriction / preterm delivery / gestational age at delivery / small for gestational age, (5) birth difficulties, (6) maternal age and sex of the child, (7) child second-hand smoke exposure or other environmental pollution exposure, (8) child injury, (9) paternal/partner psychiatric disorder or symptoms, (10) further antidepressant exposure through breastfeeding, (11) breastfeeding and (12) maternal and/or paternal IQ. We compiled this list of factors potentially related to the outcomes of interest by a brief literature review. We weighted depression or anxiety severity more highly than other factors in the comparability section because a failure to account for maternal depression in non-exposed groups has been the limitation of previous reviews. For the outcome section, we removed the second item ‘Was follow-up long enough for outcomes to occur?’ as this formed part of our inclusion criteria. The total score for our modified scale was 8, and we considered a score ≥ 6 that adjusted for severity of pre- and/or post-natal depression/anxiety to be of a lower risk of bias, and all other studies to have a higher risk of bias.

### Data transformation

#### Sample characteristics

Not all data was reported in the format we required to assess prevalence and between-group differences in sample characteristics. Where possible, we calculated the clinical and demographic characteristics of the sample and each exposure group and tested for differences in those characteristics.

#### Outcomes

Many of the included studies compared outcomes from each of the groups of interest in this review: ‘depressed, exposed’ and ‘depressed, non-exposed’ to a third (non-depressed, non-exposed) group which was not of interest to this review. In these cases, we re-calculated the difference between the depressed, exposed and depressed, non-exposed groups. We did not extract estimates where the data for the non-depressed, non-exposed group could not be separated out. To standardise the low birth weight outcomes, we computed the log odds ratio and its standard error from odds ratios/hazard ratios (computed from proportions if necessary) and their variance. One study (Oberlander et al. 2006) reported mean difference in incidence of low birth weight of a propensity-score matched sample but not the absolute incidence rate. In this case, we assumed that the overall incidence rate of the exposed group was similar to that reported for the exposed group in the non-propensity matched sample and used this to calculate the log odds ratio. Computing the z-statistic from the mean and confidence interval reported resulted in a corresponding *P* value of 0.011, which was similar to the *P* = 0.02 reported for the estimate of the mean difference in the matched sample, indicating our assumption was reasonable. For the neurodevelopmental outcomes, we standardised binary outcomes as reported above, and computed the standardised mean difference (effect size) from any continuous outcomes where possible.

### Data synthesis

#### Narrative synthesis

We report a narrative synthesis of evidence and present standardised results.

#### Meta-analysis

We planned to conduct meta-analyses of similar studies with similar outcomes that we had assessed as having a lower risk of bias (see “Quality appraisal” section). We did not perform any meta-analyses because no studies examining low birth weight met these criteria, and the two studies examining later outcomes that did meet the criteria examined different outcomes.

## Results

**Fig. 1:**
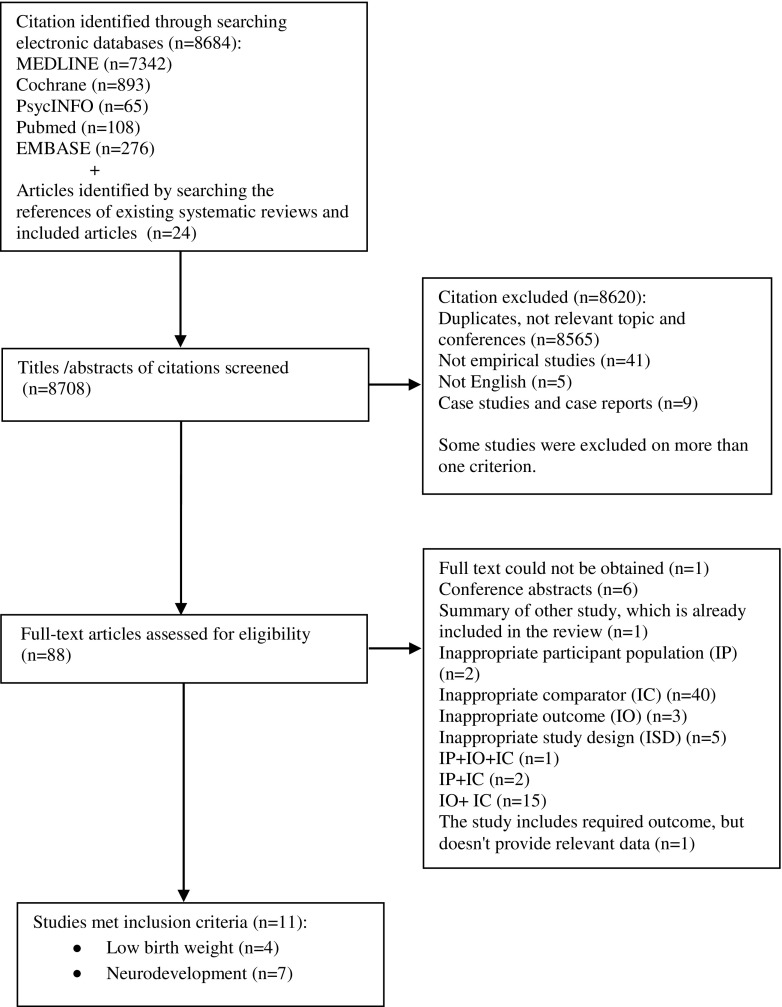
Flowchart for selection of studies included in the systematic review

A total of 8708 records were retrieved, of which 88 full-text articles were assessed for eligibility, and 11 were included in the review: four cohort studies reporting a low birth weight outcome and seven cohort studies reporting a neurodevelopmental outcome (Fig. 1).

### Maternal antidepressant use in pregnancy and offspring’s LBW

Extracted study characteristics, analyses and reported results are presented in Online Resource Tables S1a and S1b. There were two cohorts assembled from registries and data linkage from Canada (Oberlander et al. 2006) and Denmark (Jensen et al. 2013) and two prospective cohorts that recruited in the Netherlands (El Marroun et al. 2012) and Norway (Nordeng et al. 2012). All women were studied between 1996 and 2006. All studies excluded multiple births.

#### Exposure

All four studies examined selective serotonin reuptake inhibitors (SSRIs) and two also included other classes of antidepressants: one that reported specific SSRI exposure (fluoxetine, citalopram/escitalopram, paroxetine, sertraline and fluvoxamine), also included tricyclic antidepressants (TCAs) and other antidepressants (Nordeng et al. 2012); the other examined SSRIs and newer and older antidepressants without specifying them (Jensen et al. 2013). One study excluded venlafaxine (a serotonin-norepinephrine reuptake inhibitor, SNRI) because it was only used in combination with other non-SSRIs in the study population (Oberlander et al. 2006). The other did not specify which SSRIs were studied (El Marroun et al. 2012). Online Resource Table S1a.

The two data linkage studies ascertained exposure by redeemed prescriptions: the two prospective cohorts by self-reported use. Three studies, one register-based (Jensen et al. 2013) and two self-reported (El Marroun et al. 2012; Nordeng et al. 2012), considered the exposure period of the entire pregnancy but the Canadian register-based study as filling a prescription at least 49 days after the date of conception (Oberlander et al. 2006). Despite this restriction, the prevalence of antidepressant use in the entire cohort was much higher in the Canadian register-based study (2.3% in 1998 rising to 5.0% in 2001) (Oberlander et al. 2006) compared with 1.1 to 1.3% in the other three studies. Non-exposure in three studies was classified as no prescription redemption or use during pregnancy (El Marroun et al. 2012; Jensen et al. 2013; Oberlander et al. 2006); in a fourth study, the non-exposed group consisted of women who had used an AD in the 6 months prior to pregnancy but not during pregnancy (Nordeng et al. 2012).

#### Maternal mental health

No study examined anxiety. The presence of depression diagnostic codes in the medical record was used to define both exposed and non-exposed groups in the two data linkage studies: one covering the pregnancy period and the previous year (14% prevalence in whole cohort) (Oberlander et al. 2006) and one during the pregnancy only (0.6% prevalence) (Jensen et al. 2013). Thresholds of self-reported depressive symptoms on scales were used to indicate disorder in the two prospective cohorts: one a score of more than 2 on the Hopkins Symptom Checklist-5 (SCL-5) at 17 weeks gestation (6.5% prevalence) (Nordeng et al. 2012) and one a score of more than 0.75 on the 6-item depression scale of the Brief Symptom Inventory at an average 20.6 weeks gestation (prevalence not reported) (El Marroun et al. 2012). In these two studies, the scales were used to classify women in the non-exposed group, but not in the exposed group. None of the studies provided information on other treatments provided in either group. Online Resource Table S1a.

#### Outcome

Low birth weight was defined as < 10th percentile for gestational age in the two data linkage studies (Jensen et al. 2013; Oberlander et al. 2006). In the other two prospective cohorts (El Marroun et al. 2012; Nordeng et al. 2012), low birth weight was defined as smaller than 2500 g, but both studies adjusted for gestational age. All four studies ascertained birth weight from medical records. Online Resource Table S1a.

#### Characteristics of included participants

One study (Jensen et al. 2013) did not report the women’s characteristics in mutually exclusive exposure groups. There were between-exposure group differences in key socio-demographic features in each of the other three studies. In an unadjusted study (Oberlander et al. 2006) reporting few demographic data, exposed women were older, had more prenatal healthcare visits and were more likely to have subsidised prescriptions (an indicator of disadvantage); women were matched on these (and other) characteristics in the propensity-matched sample from this cohort. Exposed women in one study (Nordeng et al. 2012) were more likely to have less education and less likely to be married (indicators of disadvantage), were more likely to smoke and have been hospitalised during pregnancy. Conversely, in the Dutch study (El Marroun et al. 2012), exposed women were older and more likely to be Dutch but had more markers of advantage compared to the non-exposed group, having higher levels of education and income. Online Resource Table S1b.

#### Severity of mental health problems

Severity of depressive symptoms was reported as higher in the exposed group in one study (Nordeng et al. 2012) and higher in the non-exposed group in another (El Marroun et al. 2012). One study in which women in both groups had diagnoses did not present further data on symptom severity (Jensen et al. 2013). The fourth study indicated that the exposed group had more psychiatric health service use, but did not present symptom severity data (Oberlander et al. 2006). Online Resource Table S1b.

#### Analysis and adjustments

Only one study adjusted their regression analysis for the severity of depressive symptoms (Nordeng et al. 2012), and one other undertook propensity matching that included depressive history as noted from health service records (Oberlander et al. 2006). Three of the studies adjusted for smoking during pregnancy (El Marroun et al. 2012; Jensen et al. 2013; Nordeng et al. 2012), and one also for maternal drinking during pregnancy (El Marroun et al. 2012). Two studies adjusted for other drug exposures: use of antiepileptics, antipsychotics, other medicine (Jensen et al. 2013) and benzodiazepines (El Marroun et al. 2012). One study used two methods of analysis (Oberlander et al. 2006). In the first, they drew propensity score-matched samples, matching on some socio-demographic factors, mental health including service use, TCAs and antipsychotic prescriptions, but not smoking or drinking during pregnancy. It was unclear how this propensity-matched sample was analysed. These authors also reported an unadjusted estimate analysing the whole cohort. Women using non-SSRI antidepressants, benzodiazepines and antipsychotics were excluded from the unadjusted analysis. The two studies reporting low birth weight as an outcome adjusted for gestational age (El Marroun et al. 2012; Nordeng et al. 2012). Online Resource Table S1b.

#### Quality assessment

All the studies scored between 5 and 6 out of 8 on the modified NOS quality assessment scale for cohort studies (Online Resource Table S2), but none met our criteria for lower risk of bias. All studies scored relatively highly on the selection section and outcome criteria, reflective of study designs that were broadly representative of pregnant women, selected all women using the same method and had an outcome that was ascertained using routine records. No study scored the maximum two points on the section ‘Assessing comparability of the exposure groups’ because none controlled for all the factors we deemed necessary to be comparable between the exposed and non-exposed groups. One study reporting two different analysis samples (Oberlander et al. 2006) scored zero on the comparability section.

#### Results

We present standardised effect ratios on the log odds ratio scale (Table 1). The study that controlled for depressive symptoms (Nordeng et al. 2012) did not report a difference in LBW between exposure groups. The large unadjusted study (Oberlander et al. 2006) and an adjusted study (El Marroun et al. 2014) also reported finding no evidence of effect. Two studies (Jensen et al. 2013; Oberlander et al. 2006) indicated statistically significant effect ratios, but although one matched exposure groups on psychiatric-related health service use (Oberlander et al. 2006), neither controlled for depression severity.

**Table 1:** Results for low birth weight

Study, antidepressants studied	Effect ratio	95% CI	*N*	Adjustments/*exclusions*/***stratification***
Nordeng et al. (2012), SSRI	0.67	0.29, 1.52	1747	a, b, d, h, g, j, i, l
Jensen et al. (2013), AD	**1.42**	**1.16**, **1.73**	3966	c, d, f, g, o
El Marroun et al. (2012), SSRI	1.72	0.73, 4.07	669	c, d, e, f, g, h, j, k, m
Oberlander et al. (2006), non-PS SSRI	1.05	0.87, 1.28	15,685	*f*
Oberlander et al. (2006), PS SSRI	**1.69**	**1.14**, **2.52**	1622	c, f, p

Estimates in bold are statistically significant. Effect ratio is on log odds scale. a. Maternal mental health pre-birth, b. maternal age, c. socio-economic status, d. smoking in pregnancy, e. alcohol in pregnancy, f. other psychoactive drug and/or medication use during pregnancy, g. sex of child, h. gestational age, i. illness/disease during pregnancy, j. parity, k. ethnicity, l. folic acid use, m. body mass index, o. calendar year of delivery, p. psychiatric-related health service use

*PS* propensity score-matched sample, *SSRI* selective serotonin reuptake inhibitors, *AD* antidepressant, *CI* confidence interval

### Maternal antidepressant use in pregnancy and offspring’s neurodevelopmental and neurobehavioural outcomes

Extracted study characteristics, analyses and reported results are presented in Online Resource Tables S3a and S3b. Data relating to the two groups of interest (exposed, depressed/anxious and non-exposed) in the seven included studies were gathered from prospective cohorts: one each from the Netherlands (El Marroun et al. 2014) and Canada (Nulman et al. 2012), three from the USA (Casper et al. 2003; Santucci et al. 2014; Suri et al. 2011) and two using data from the Danish National Birth Cohort (Pedersen et al. 2010, 2013). Analysed sample sizes ranged from 44 to 604 (*N* = 31 to 294 exposed, *N* = 13 to 376 non-exposed), with median sample sizes of *N* = 69 exposed and *N* = 54 non-exposed.

#### Exposure

Two studies investigated SSRIs (Casper et al. 2003; El Marroun et al. 2014); two examined SSRIs and venlafaxine (Nulman et al. 2012; Santucci et al. 2014); two examined SSRIs, TCAs and other antidepressants or combinations (Pedersen et al. 2010, 2013); and one did not specify the type of antidepressants but found the majority exposed to sertraline and fluoxetine (Suri et al. 2011). The exposure period was defined as ‘any use in pregnancy’ by six studies and as use in > 50% of the pregnancy in one (Suri et al. 2011). Only one study constructed the non-exposed group from women who discontinued antidepressants prior to pregnancy (Nulman et al. 2012). In all studies, exposure was ascertained by self-report. For the two studies that recruited a population cohort, the prevalence of exposure was calculated at 0.5% (Pedersen et al. 2010) and 1.17% (El Marroun et al. 2014). Online Resource Table S3a.

#### Maternal mental health

All seven studies examined depression, or depressive symptoms, and none anxiety. One study used a threshold of > 0.75 on the depression scale of the Brief Symptom Inventory (administered at 21 weeks gestation) to indicate clinically relevant depressive symptoms in the unexposed group (El Marroun et al. 2014). The exposed group was defined on AD exposure only. The two studies using the Danish National Cohort (Pedersen et al. 2010, 2013) reported using responses to four questions about psychiatric disorders and care asked at 17 and 32 weeks gestation to determine depression, but it was not clear how they were used, or whether the same criteria were applied to the exposed group. One study used the women’s psychiatrist’s diagnoses of depressive episodes to define both the exposed and non-exposed groups (Nulman et al. 2012). Three studies defined disorder in both the exposed and non-exposed groups as diagnoses ascertained via a structured clinical interview during pregnancy: major depressive disorder in two (Santucci et al. 2014; Suri et al. 2011) and any DSM-IV Axis I disorder in one (Casper et al. 2003). For the two studies that recruited a population cohort, the prevalence of disorder was calculated at 1.1% (assuming all exposed were depressed) (Pedersen et al. 2010) and 14% (El Marroun et al. 2014). One study reported all women also received psychotherapy (Casper et al. 2003), another indicated that depression in the non-exposed group was untreated (Nulman et al. 2012) and the remaining studies did not report whether the non-exposed group received any alternative treatment for depression (El Marroun et al. 2014; Pedersen et al. 2010, 2013; Santucci et al. 2014; Suri et al. 2011). Online Resource Table S3a.

#### Outcomes

The median number of outcomes in a study was 9 (min *N* = 5, max *N* = 15). Many of the multiple outcomes were due to analysing estimates from instrument subscales. Five studies measured at least one outcome of abnormal development as a threshold of a measurement scale: pervasive development problems (El Marroun et al. 2014), behaviour (Nulman et al. 2012; Pedersen et al. 2013), behavioural development (Santucci et al. 2014), developmental milestones (Pedersen et al. 2010) and attention deficit hyperactivity disorder (ADHD) and comorbid disorders (Nulman et al. 2012). The remaining outcomes were measured as mean scores on a scale that could be broadly categorised as follows: autistic symptoms and specific autistic symptoms (El Marroun et al. 2014), behaviour (Nulman et al. 2012; Pedersen et al. 2013), IQ (Nulman et al. 2012), ADHD and comorbid disorders (Nulman et al. 2012), neonate behaviour (Suri et al. 2011), mental development (Casper et al. 2003; Santucci et al. 2014), psychomotor development (Casper et al. 2003; Santucci et al. 2014), motor quality (Casper et al. 2003) and behavioural development (Casper et al. 2003). Fifty percent (*N* = 34) of the outcomes were assessed by an independent rater such as a psychologist, and 50% by a parent (usually the mother) scoring the child on a scale. Five studies measured some or all of the outcomes at multiple time points (El Marroun et al. 2014; Pedersen et al. 2010; Santucci et al. 2014; Suri et al. 2011). The oldest age of assessment in any one study ranged from 6–8 weeks to 6 years 11 months. Online Resource Table S3a.

#### Characteristics of included participants

Most studies reported some differences in characteristics between exposure groups. One (El Marroun et al. 2014) reported that exposed women were older, had more education, were more likely to be Dutch, were more likely to have drunk alcohol during pregnancy and were more likely having given birth to girls than non-exposed women. Another (Pedersen et al. 2013) reported no differences on key characteristics but we could not ascertain whether there were differences in maternal age and caffeine intake as data were incompletely reported. Children in the non-exposed group were older at the time of assessment in one study (Nulman et al. 2012), and had a longer mean gestational age in another (Suri et al. 2011). One study (Pedersen et al. 2010) reported that exposed women were older and had higher educational attainment; however, the data presented included those for whom the child’s outcome was missing so we could not tell whether this was the case for the analysed sample. Women in the exposed group in one study (Santucci et al. 2014) were more likely to be White, have completed university and be married or cohabiting, and exposed children in another (Casper et al. 2003) were more likely to have a mother taking an SSRI while breastfeeding and had lower APGAR scores than non-exposed children. Online Resource Table S3b.

#### Severity of mental health problems

Severity of prenatal depressive symptoms was reported as higher in the non-exposed group in three studies (El Marroun et al. 2014; Pedersen et al. 2010, 2013) and higher in both the non-exposed group and the women exposed to SSRIs, versus the women exposed to venlafaxine, in another (Nulman et al. 2012). Three studies (Casper et al. 2003; Santucci et al. 2014; Suri et al. 2011) did not find a statistically significant difference in severity symptoms between exposure groups. Online Resource Table S3b.

Severity of depressive symptoms measured at some point after delivery was reported as higher in the non-exposed group in two studies (El Marroun et al. 2014; Pedersen et al. 2010). No between-group differences in symptom severity were detected in two studies (Nulman et al. 2012; Suri et al. 2011), and differences were not measured or reported in three (Casper et al. 2003; Pedersen et al. 2013; Santucci et al. 2014). One study (Pedersen et al. 2013) noted that they found no difference in the proportion of women who met DSM-IV criteria for major depression, but women in the exposed group were more likely to be on medical treatment for depression since the delivery.

#### Analysis and adjustments

Three studies adjusted some analyses for depressive symptoms at some time after the child was born (El Marroun et al. 2014; Pedersen et al. 2010, 2013), and one for symptoms both during pregnancy and at some time after birth (Suri et al. 2011). Smoking during pregnancy and markers of socio-economic status were adjusted for in two studies (El Marroun et al. 2014; Pedersen et al. 2013) and results stratified by maternal smoking and drinking during pregnancy in a third (Pedersen et al. 2010). Three studies adjusted for maternal age (El Marroun et al. 2014; Pedersen et al. 2010, 2013), three the sex of the child (El Marroun et al. 2014; Pedersen et al. 2010, 2013) and two gestational age at birth (El Marroun et al. 2014; Suri et al. 2011). Five studies excluded women taking other psychotropic or teratogenic medications during pregnancy from the analysis (Nulman et al. 2012; Pedersen et al. 2010; Santucci et al. 2014; Suri et al. 2011), one did not adjust the analysis for the higher usage of benzodiazapines in the exposed group (El Marroun et al. 2014) and one did not report or adjust for other medication during pregnancy (Casper et al. 2003). One study stratified results by exposure window and type of antidepressant (Pedersen et al. 2010). Most studies reported using multivariable linear (El Marroun et al. 2014; Pedersen et al. 2010, 2013) or logistic ( Pedersen et al. 2010, 2013) regression or analysis of covariance (Casper et al. 2003; Suri et al. 2011) to analyse outcomes, but unadjusted proportions (Nulman et al. 2012; Santucci et al. 2014) and unadjusted means (Casper et al. 2003; Santucci et al. 2014) were also reported. Online Resource Table S3b.

#### Quality assessment

Only two studies met our criteria for lower risk of bias (El Marroun et al. 2014; Suri et al. 2011) (Online Resource Table S4). Only one study (Suri et al. 2011) adjusted estimates for pregnancy depression severity, and four for post-delivery severity (El Marroun et al. 2014; Pedersen et al. 2010, 2013; Suri et al. 2011). Two adjusted for some marker of socio-economic status (El Marroun et al. 2014; Pedersen et al. 2013), and these, along with two more (Pedersen et al. 2010; Suri et al. 2011), controlled for at least another two of our pre-defined potential confounders. Three studies (Casper et al. 2003; Nulman et al. 2012; Santucci et al. 2014) did not adjust their analyses for any potential confounders although one excluded users of benzodiazepines or any US FDA pregnancy class D or X drugs (Santucci et al. 2014) and another excluded users of known teratogens and polytherapy for depression (Nulman et al. 2012). Only one study (Suri et al. 2011) scored the maximum two points on the ‘Outcome’ section, with others losing points mainly because child outcomes were reported by the parents and not an independent observer, or the method of ascertainment was not described.

#### Results

Results are reported grouped by child age (Tables 2, 3, 4 and 5).

**Table 2:** Results for neonate behaviour

Study, antidepressants studied	Child age at testing (weeks)	Outcome	Effect size	95% CI	*N*	Adjustments/*exclusions*/***stratification***
Suri et al. (2011), NR (most exposures sertraline and fluoxetine)	6–8	1a. Habituation	**0.81**	**0.14**, **1.48**	46	*g*
		1b. Orientation	− 0.38	− 1.03, 0.27	46	*g*
		1c. Motor	− 0.43	− 1.09, 0.22	46	*g*
		1d. Defence	0.19	− 0.46, 0.84	46	*g*
		1e. Range of state	− 0.11	− 0.76, 0.54	46	*g*
		1f. Regulation of state	0.17	− 0.48, 0.82	46	*g*
		1g. Autonomic stability	− 0.27	− 0.92, 0.38	46	*g*
		h. Reflexes	0.30	− 0.35, 0.95	46	*g*

Estimates in bold are statistically significant. a. Maternal mental health pre-birth, b. maternal mental health at some point after delivery, c. maternal age, d. socio-economic status, e. smoking in pregnancy, f. alcohol in pregnancy, g. other psychoactive drug and/or medication use during pregnancy, h. sex of child, i. gestational age, j. age of child at testing, k. APGAR scores, l. breastfeeding, m. problems during pregnancy, n. mother-child connection, o. postnatal difficulties, p. ethnicity, q. exposure window

*NR* not reported, *CI* confidence interval

**Table 3:** Results for infant and toddler development

Study, antidepressants studied	Child age at testing (weeks)	Outcome	Effect ratio	95% CI	*N*	Adjustments/*exclusions*/***stratification***
Santucci et al. (2014), SSRI and venlafaxine	12	1b. BRS subscale—attention/arousal	0.54	0.14, 2.09	53	*g*
	78	1a. Behavioural rating scale—total score	2.38	0.25, 23.2	37	*g*
		1c. BRS subscale—orientation/engagement	3.00	0.32, 28.4	37	*g*
		1d. BRS subscale—emotional regulation	1.41	0.30, 6.68	37	*g*
		1e. BRS subscale—motor quality	2.29	0.49, 10.6	37	*g*
Pedersen et al. (2010), AD	78	1a. Gross-motor—going up stairs with support	1.00	0.49, 2.03	478	b, c, ***e***, ***f***, *g*, h, j, l, m, n, o, ***q***
		1c. Fine motor—taking off socks and shoes when asked to	1.10	0.71, 1.71	483	b, c, ***e***, ***f***, *g*, h, j, l, m, n, o, ***q***
		1d. Fine motor—drinking from ordinary cup without help	3.40	0.67, 17.3	482	b, c, ***e***, ***f***, *g*, h, j, l, m, n, o, ***q***
		1e. Attention—being occupied alone for ≥ 15 min	1.20	0.75, 1.93	486	b, c, ***e***, ***f***, *g*, h, j, l, m, n, o, ***q***
		1f. Cognition—bringing things when told to	0.80	0.27, 2.37	478	b, c, ***e***, ***f***, *g*, h, j, l, m, n, o, ***q***
		1g. Cognition—making marks on table or paper	1.30	0.57, 2.97	490	b, c, ***e***, ***f***, *g*, h, j, l, m, n, o, ***q***
		1h. Cognition—aligning picture correctly	1.00	0.69, 1.45	460	b, c, ***e***, ***f***, *g*, h, j, l, m, n, o, ***q***
		1i. Language—using word-like sounds to tell what s/he wants	1.40	0.61, 3.21	492	b, c, ***e***, ***f***, *g*, h, j, l, m, n, o, ***q***
		1j. Language—mentioning > 25 names of different things	1.70	0.94, 3.07	492	b, c, ***e***, ***f***, *g*, h, j, l, m, n, o, ***q***
		1k. Language—using 2-word sentences	1.20	0.83, 1.74	467	b, c, ***e***, ***f***, *g*, h, j, l, m, n, o, ***q***
		1l. Failed ≥ 1 milestone	2.10	0.93, 4.75	492	b, c, ***e***, ***f***, *g*, h, j, l, m, n, o, ***q***
			Effect size			
Santucci et al. (2014), SSRI and venlafaxine	78	2. Mental development	− 0.16	− 0.86, 0.55	38	*g*
Santucci et al. (2014), SSRI and venlafaxine	78	3. Psychomotor development	− 0.64	− 1.35, 0.08	38	*g*
Pedersen et al. (2010), AD	–	1b. Age at which child walked without support (difference in days)	**13.6**	**4.0**, **23.3**	NR	b, c, ***e***, ***f***, *g*, h, j, l, m, n, o, ***q***
Pedersen et al. (2010), SSRI	–		**15.2**	**4.6**, **25.9**	NR	b, c, ***e***, ***f***, *g*, h, j, l, m, n, o, ***q***
Pedersen et al. (2010), TCA	–		11.9	− 12.0, 35.8	NR	b, c, ***e***, ***f***, *g*, h, j, l, m, n, o, ***q***
			Other metric			
Casper et al. (2003), SSRI	26–173	1a. Behavioural rating scale	*F* = 2.57, *P* = 0.12		44	k
		1b. BRS subscale—attention/arousal	*F* = 1.2, *P* = 0.31		44	k
		1c. BRS subscale—orientation/engagement	*F* = 0.02, *P* = 0.88		44	k
		1d. BRS subscale—emotional regulation	*F* = 0.07, *P* = 0.79		44	k
		1e. BRS subscale—motor quality	***F*** **= 4.02**, ***P*** **= 0.05**		44	k
Casper et al. (2003), SSRI	26–173	3. Mental development	*F* = 2.12, *P* = 0.15		44	k
		2a. Gross motor movement	*F* = 2.01, *P* = 0.17		44	k
		2b. Fine motor movement	*F* = 2.22, *P* = 0.15		44	k
		2c. Control of movement	*F* = 0.55, *P* = 0.46		44	k
		2d. Tremulousness	*F* = 3.37, *P* = 0.08		44	k
		2e. Slow and delayed movement	*F* = 0.06, *P* = 0.81		44	k
		2f. Frenetic movement	*F* = 2.14, *P* = 0.15		44	k
		2g. Hypertonicity	*F* = 0.74, *P* = 0.40		44	k
		2h. Hypotonicity	*F* = 0.05, *P* = 0.83		44	k
		4. Psychomotor development	***F*** **= 5.55**, ***P*** **= 0.02**		44	k

Estimates in bold are statistically significant. Effect ratio is on log odds scale. a. Maternal mental health pre-birth, b. maternal mental health at some point after delivery, c. maternal age, d. socio-economic status, e. smoking in pregnancy, f. alcohol in pregnancy, g. other psychoactive drug and/or medication use during pregnancy, h. sex of child, i. gestational age, j. age of child at testing, k. APGAR scores, l. breastfeeding, m. problems during pregnancy, n. mother-child connection, o. postnatal difficulties, p. ethnicity, q. exposure window

*SSRI* selective serotonin reuptake inhibitors, *AD* antidepressants generally, *CI* confidence interval

**Table 4:** Results for child behavioural outcomes

Study, antidepressants studied	Child age at testing	Outcome	Effect ratio	95% CI	*N*	Adjustments/*exclusions*/***stratification***
El Marroun et al. (2012), SSRI	18 months, 3 and 6 years	1. Pervasive Developmental Problems	1.33	0.71, 2.50	445	c, d, e, h, i
Nulman et al. (2012), venlafaxine	3–7 years	2a. Abnormal Total Difficulties	1.34	0.36, 5.02	116	*g*
Nulman et al. (2012) SSRI			1.59	0.44, 5.76	116	*g*
Pedersen et al. (2013), AD	4–5 years	1a. Abnormal Total Difficulties	1.30	0.41, 4.11	225	c, d, e, f, *g*, h
		1b. Abnormal Emotional subscale	1.60	0.48, 5.34	225	c, d, e, f, *g*, h
		1c. Abnormal Conduct subscale	0.60	0.29, 1.25	225	c, d, e, f, *g*, h
		1d. Abnormal Hyperactivity subscale	1.80	0.59, 5.50	225	c, d, e, f, *g*, h
		1e. Abnormal Peer subscale	0.90	0.18, 4.41	225	c, d, e, f, *g*, h
		1f. Abnormal Prosocial subscale	0.50	0.17, 1.46	225	c, d, e, f, *g*, h
			Effect size			
Pedersen et al. (2013), AD	4–5 years	2a. Total Difficulties score	− 0.70	− 1.80, 0.40	225	c, d, e, f, g, h
		2b. Emotional subscale score	− 0.30	− 0.70, 0.10	225	c, d, e, f, *g*, h
		2c. Conduct subscale score	− 0.10	− 0.50, 0.30	225	c, d, e, f, *g*, h
		2d. Hyperactivity subscale score	− 0.20	− 0.70, 0.40	225	c, d, e, f, *g*, h
		2e. Peer subscale score	− 0.10	− 0.40, 0.20	225	c, d, e, f, *g*, h
		2f. Prosocial subscale score	0.10	− 0.40, 0.50	225	c, d, e, f, *g*, h

Estimates in bold are statistically significant. Effect ratio is on log odds scale. a. Maternal mental health pre-birth, b. maternal mental health at some point after delivery, c. maternal age, d. socio-economic status, e. smoking in pregnancy, f. alcohol in pregnancy, g. other psychoactive drug and/or medication use during pregnancy, h. sex of child, i. gestational age, j. age of child at testing, k. APGAR scores, l. breastfeeding, m. problems during pregnancy, n. mother-child connection, o. postnatal difficulties, p. ethnicity, q. exposure window

*SSRI* selective serotonin reuptake inhibitors, *AD* antidepressants generally, *CI* confidence interval

**Table 5:** Results for child autistic symptoms, ADHD and comorbid disorders

Study, antidepressants studied	Child age at testing (years)	Outcome	Effect size	95% CI	*N*	Adjustments/*exclusions*/***stratification***
Child autistic symptoms						
El Marroun et al. (2012), SSRI	6	2. Autistic symptoms	0.10	− 0.32, 0.52	272	b, c, d, e, h, i, p
		3a. Social cognition	0.08	− 0.36, 0.52	272	b, c, d, e, h, i, p
		3b. Social communication	0.12	− 0.36, 0.60	272	b, c, d, e, h, i, p
		3c. Autistic mannerisms	0.08	− 0.31, 0.47	272	b, c, d, e, h, i, p
			Effect ratio			
Child ADHD and comorbid disorders						
Nulman et al. (2012), venlafaxine	3–7	4a. Total problems	4.65	0.53, 41.1	116	*g*
SSRI			**11.4**	**1.42**, **91.8**	116	*g*
Venlafaxine		4b. DSM total symptoms	0.85	0.28, 2.61	116	*g*
SSRI			0.85	0.28, 2.61	116	*g*

Estimates in bold are statistically significant. Effect ratio is on log odds scale. a. Maternal mental health pre-birth, b. maternal mental health at some point after delivery, c. maternal age, d. socio-economic status, e. smoking in pregnancy, f. alcohol in pregnancy, g. other psychoactive drug and/or medication use during pregnancy, h. sex of child, i. gestational age, j. age of child at testing, k. APGAR scores, l. breastfeeding, m. problems during pregnancy, n. mother-child connection, o. postnatal difficulties, p. ethnicity, q. exposure window

*SSRI* selective serotonin reuptake inhibitors, *CI* confidence interval

#### Neonate behaviour

The results from one study with a lower risk of bias (Suri et al. 2011) indicated few differences in neonate behaviour measured by the BNBAS between exposure groups, except for a mean score difference in habituation (Table 2). The authors reported (in narrative) no effect by exposure group after adjusting for gestational age at delivery, mean and maximum HDRS (depressive symptom) scores in pregnancy and 4 and 8 weeks after delivery, and sex of the child; however, these models also included the non-depressed, non-exposed group.

#### Infant and toddler development

Three studies, all with higher risk of bias, measured infant and toddler development (Table 3). Two of the 15 measurements made by one study (Casper et al. 2003) (BRS subscale Motor Quality and Psychomotor development, adjusted for 5-min APGAR score) indicated statistically significant worse development for children age 26–173 weeks exposed to SSRIs. One study (Pedersen et al. 2010) noted a statistically significant difference of 13.6 days in the retrospectively reported age at which the child first walked without support for children exposed to antidepressants (adjusted for a range of confounders) and a larger difference (28.9 days) for women exposed in the second/third trimester after stratification (for antidepressants overall and for SSRIs). They found no other between-group differences in the other 10 items measured, including after stratification for exposure window. The third study (Santucci et al. 2014) noted no between-group differences for the seven items they measured (unadjusted analyses).

#### Child behavioural outcomes

None of the three studies (El Marroun et al. 2014; Nulman et al. 2012; Pedersen et al. 2013) only one (El Marroun et al. 2014) with a lower risk of bias, reporting a total of 15 behavioural outcomes, found a statistically significant difference by exposure group (Table 4).

#### Child autistic symptoms

There were no differences between exposure groups in symptoms of autism reported by the mother on the SRS at age 6 for the one study that reported these outcomes (El Marroun et al. 2014) (lower risk of bias) (Table 5).

#### ADHD and comorbid disorders

The one study (Nulman et al. 2012) (higher risk of bias) examining this outcome found a statistically significant higher proportion of 3–7-year-old children with a clinically significant total problems score on the Conners’ Parent Rating Scale (parent reported, unadjusted) exposed to SSRIs, but not for the children who were venlafaxine-exposed (Table 5). They found no between-group variation in the DSM total symptom scores.

## Discussion

Untreated common mental disorders and symptoms during pregnancy pose risks to offspring (Gentile 2015; Kingston et al. 2012). Therefore, to answer a clinically relevant question, the effect of in utero antidepressant exposure on children should be ascertained against the effects of common mental disorders during pregnancy. Previous systematic reviews have been limited by comparing exposed children with children of healthy women. We conducted a systematic review of observational studies examining birth weight and development outcomes for children exposed to antidepressants in utero compared to children of women with common mental disorders, or symptoms of common mental disorders, but no antidepressant exposure. Despite selecting only those studies with such a control group, few analyses were controlled for depressive symptom *severity* between exposure groups, raising concerns about selection bias. This, along with other design limitations and sources of bias, limits the conclusions we can draw from the synthesis.

### Non-exposed comparators

Only two studies out of the 11 included in our review constructed the non-exposed comparator group solely from women who were exposed in the months prior to pregnancy but not during pregnancy. This situation most closely represents the clinical problem, namely should women needing to take antidepressants, and considering pregnancy, discontinue them prior to pregnancy, that is will the effect of not taking them outweigh the effect of non-medically treated symptoms? Antidepressants are not a first-line therapy for mild to moderate common mental disorder, and women who never take antidepressants may, on average, have less severe symptoms, which potentially could exert fewer biological effects. Alternatively, women could refuse medical treatment for a moderate to severe episode. Constructing the comparison group from a non-antidepressants-using cohort is therefore of limited value unless analyses account for any potential difference in symptom severity. We acknowledge that a definitive controlled trial randomising women to either discontinue antidepressants prior to conception or continue them through pregnancy is both unethical and largely unfeasible. We consider, however, that much more could be done to attempt to limit differences and control for differential effects, and also believe that a preference trial variant (Torgerson and Sibbald 1998) may be both desirable and possible to conduct in the maternal setting.

### Outcomes: low birth weight

We found only limited evidence of lower birth weight in children exposed to antidepressants in two studies which had a higher risk of bias and did not control for depressive symptom severity. These studies were both retrospective: one a data linkage study and one a register-based cohort. In an older review, Ross et al. (2013) examined a similar question, finding no evidence of effect. Only one of our included studies overlapped those reviewed by Ross et al. due to a variation in exclusion criteria and dates searched. Together, these syntheses indicate that there is currently little evidence to indicate that antidepressant use in pregnancy causes children to be born with lower birth weight accounting for gestational age. Depression itself has been associated with LBW (Grote et al. 2010), but basic science studies also confirm the cross placental passage of SSRIs and the subsequent effects on vascularisation which could result in LBW (Wessler et al. 2007). Therefore, future studies should continue to analyse the link.

### Outcomes: neurodevelopment and neurobehaviour

Out of 59 child neurodevelopmental effect estimates we examined, only five (8.5%) showed evidence of a statistically significant effect, which could have been due to type I error, or chance false positive. All three of the studies reporting a statistically significant effect were assessed as having a higher risk of bias. The single study of neonate behaviour was of lower risk of bias and found an effect in only one out of eight outcomes (12.5%). While all the studies were of a prospective design, many were very small and likely underpowered. Even in the case of the studies demonstrating significant effects, their clinical importance can be questioned. For example, there is a large normal range of time it takes for a child to walk unsupported, within which a difference of 13.6 days may be a reflection of this variation rather than an increase in the delay of onset of walking. The results should thus be interpreted with caution. Importantly, serotonin has diverse functions in utero to guide foetal development (Bourke et al. 2014). As documented in animal models, there are also natural processes involving a switch from a placental serotonin to endogenous foetal serotonin (Bonnin et al. 2011) during development, and thus, any disruptions during critical times of foetal development may potentially have long-term effects particularly for the foetal brain. Therefore, future studies should continue the exploration of the effect of antidepressants on neurodevelopmental outcomes.

### Future research

To overcome the limitations we have uncovered in our review, our main recommendation is that a study design standard is developed. This could be achieved by any range of consensus methods such as those used to generate core outcome sets (Gargon et al. 2014). We recommend this because studies examining child outcomes are typically small and outcomes rare, yet they are currently too dissimilar and/or biased to pool in meta-analyses. Based on the findings of our review, areas to consider include ascertainment, measurement and reporting of exposure, disorder and outcomes, timing of exposure, other treatments, and collection of socio-demographic data and other factors that could potentially confound any particular outcome (Bandoli et al. 2016).

In the meantime, we recommend that researchers continuing to analyse data construct two separate non-exposed comparator groups. The first is ascertained in the same way to the exposed group and varying only in that women are exposed to antidepressants in the months prior to pregnancy but have discontinued by the washout period (defined by the exposure window on the outcome) prior to conception. The second group is similarly ascertained but women have no antidepressant exposure in at least the year prior to pregnancy. Symptom severity should be measured in all groups, exposed and non-exposed, and symptom scores adjusted for in multivariate analyses. Using data about service use in place of direct measurement of symptom severity is likely to under-ascertain disorder severity for some women, as service use may not be proportionate to need particularly among disadvantaged groups. The lack of verification of accurate and comparable between-group ascertainment is a major limitation in currently available routine data and register-based linkage. Researchers constructing comparative groups using such data should consider using methods that minimise ascertainment bias such as matching exposed and non-exposed women on date and timing of diagnoses, for example, and conducting sensitivity analyses on study assumptions. Any effects of restricting the sample in this way on the generalisability of the study population should be carefully reviewed. Data on relapse following discontinuation during pregnancy are sparse and conflicting (Cohen et al. 2004, 2006; Yonkers et al. 2011), but accurate information on relapse and its effect is an important factor needed to balance the argument on risk of treatment discontinuation. Relapse in any exposure group during pregnancy should be identified, and this information analysed along with predictors of this risk such as the number of previous episodes and the start of current episode. The presentation of both bivariate and multivariate risk estimates would further our understanding about the size of effects due to variation in symptom severity. The presentation of multivariate estimates is also crucial to our ability to accurately synthesise studies, even if the addition of a particular covariate does not substantially change a point estimate in an individual study. Although anxiety can be treated with antidepressants (Howard et al. 2014), we found no studies on anxiety that matched our inclusion criteria. Further research on anxiety and its treatment in pregnancy is urgently needed.

### External generalisability

Where it could be calculated, we found variation in the prevalence of antidepressant use during pregnancy and in the prevalence of depression and depressive symptomology in whole cohorts. These differences may reflect between-country variation in guidelines for prescribing during pregnancy, and treatment success, with potential consequences for variation in which women of different clinical and/or social characteristics were selected into each exposure group. It could also reflect differences in ascertainment method (self-report vs. linked data on prescriptions) and timing of exposure windows.

### Strengths and limitations

We double checked all our extracted data and risk of bias assessments; however, only one person searched, screened and selected studies for inclusion which may have resulted in some studies being missed. Like others (Stang 2010), we did not find the NOS sensitive to limitations in study design without significant alteration; the use of another tool may have resulted in a better differentiated assessment of study quality. Due to resource limitations, we were unable to include articles published in languages other than English, which may have resulted in us not including all relevant studies.

## Conclusion

We found only very limited evidence from observational studies that birth weight and child neurodevelopment and neurobehaviour are impacted by gestational exposure to antidepressants. We were unable to conduct meta-analyses due to a high risk of bias and variation in study design. Accordingly, we cannot be certain that any effects attributed to antidepressant exposure are not reflecting underlying differences in clinical and social characteristics of women who continue antidepressants in pregnancy, compared to those who discontinue, or those who do not take them at all. Standardising how studies ascertain, measure and report exposures, disorders, outcomes and other treatments would improve our ability to accurately estimate the presence and size of effects, and ultimately provide less biased information with which to inform clinical decision-making.

## Electronic supplementary material

ESM 1 (DOCX 24.1 kb)

ESM 2 (DOCX 23.3 kb)

ESM 3 (DOCX 20.3 kb)

ESM 4 (DOCX 30.2 kb)

ESM 5 (DOCX 34.1 kb)

ESM 6 (DOCX 21 kb)
