# Supplementary material for: A systematic review of maternal antidepressant use in pregnancy and short- and long-term offspring’s outcomes
Source: Arch Womens Ment Health. 2017 Oct 12;21(2):127–40. doi: 10.1007/s00737-017-0780-3 (PMC5856864; doi:10.1007/s00737-017-0780-3)
Supplement: Supplementary file 1 — (DOCX 24.1 kb) [file 737_2017_780_MOESM1_ESM.docx]

**Table S1a: Study characteristics - low birth weight outcomes**

|  |  |  |  | **Group definition and ascertainment** | |  |  |
| --- | --- | --- | --- | --- | --- | --- | --- |
| **Study** | **Study design** | **Location, recruitment and exclusions** | **Antidepressants studied, quantity and duration of exposure** | **Exposed group** | **Unexposed group** | **Prevalence*** | **Low birth weight** |
| Oberlander et al. (2006) | Retrospective data linkage cohort | British Columbia (BC), Canada  Live births registered between 1997-2002 in the BC births registry (N=203,520) linked to hospital separation records, registry of subsidised prescriptions, and physician billing records. Deterministically linked to PharmNet records of all prescriptions dispensed by BC pharmacists.  Excluded unlinked records, date of conception >01/01/1998 & <03/26/2001, data entry errors, multiple births, those with non-SSRI AD prescriptions, benzodiazapines or antipsychotics during pregnancy; study sample size N=119,547 pregnancies. | SSRIs (excluding venlafaxine)  Most common SSRIs were paroxetine (44.7%), fluoxetine (27.2%), sertraline (25.6%), fluvoxamine (4.6%), citalopram (3.3%). | *AD exposure:*  *Definition*: filling an SSRI prescription at least 49 days after conception  *Ascertainment*: linked pharmacy records  *Mental disorder:*  *Definition*: depression diagnosis code/s during pregnancy or in the year prior to pregnancy  *Ascertainment*: one or more of the following ICD-9 depression codes in Medical Services Plan billing records: 290.2, 296, 296.1-296.6, 298, 300.4, 309, 309.1, 311, 508 | *AD exposure:*  *Definition*: No ADs, benzodiazapines or antipsychotics during pregnancy  *Ascertainment:*As exposed  *Mental disorder:*As exposed  *Ascertainment*: As exposed | *AD exposure*: 2.3% (in 1998) to 5.0% (in 2001)  *Mental disorder:* 14% (whole period) | *Definition*: <10th percentile for gestational age  *Ascertainment*: hospital birth records  *Prevalence*: 7.5% (8.1%) No prevalance given for PS matched sample |
| Jensen et al. (2013) | Retrospective register based cohort | Denmark  Pregnancies between 1996-2006 from Medical Birth Register (N=963,585)  Excluded non-live births, multiple births, gestational age at birth <22 weeks; study sample size N=673,853 pregnancies (69.9%) (672,601 children by 440,835 mothers) | SSRIs, newer AD, older AD (TCA)  Of the N=8,511 exposed, N=7,510 (88.2%) in the 1^st^ trimester, N=3,837 (45.1%) in the 2^nd^ and N=3,300 (38.7%) in 3^rd^. | *AD exposure:*  *Definition*: redeemed prescriptions of interest during pregnancy  *Ascertainment*: linked Medicinal Product Statistics database  *Mental disorder:*  *Definition*: depression diagnosis codes during pregnancy  *Ascertainment*: had one or more of the following depression diagnosis codes: ICD-8 296.09 and 296.29; ICD-10 DF32.00-DF33.99 from the Danish Psychiatric Central Register, covering inpatient and outpatient public mental health services | *AD exposure:*  *Definition*: did not redeem prescriptions of interest during pregnancy  *Ascertainment:*As exposed  *Mental disorder:*  *Definition*: As exposed  *Ascertainment*: As exposed | AD exposure: 1.3%  Mental disorder: 0.6% | *Definition*: <10th percentile of birthweight at a given gestational week (weeks 22-45)  *Ascertainment*: Medical Birth Register  *Prevalence*:  LBW at term: 9.6% (NR)  LBW pre-term: NR (NR) |
| Nordeng et al. (2012) | Prospective cohort (MoBa) | Norway  MoBa enrollees were recuited at ultrasound visit (gestational weeks 17-18) from most medical centres in Norway.  Exclusions for this study were having a pregnancy outcome outside of 2000-2006, failing to complete both pregnancy questionnaires, pregnancy outcome not available from the Medical Birth Registry of Norway, multiple births, a chromosomal abnormality in pregnancy. N=72,993 women had a pregnancy outcome between 2000-2006 with Medical Birth Registry linkage; study sample size N=63,395 women | Any drug under WHO-defined Anatomic Therapeutic Chemical Classification System (ATC) group N06A  Of N=699 exposed, N=556 (79.5%) in 1^st^ trimester, N=323 (46.2%) in 2^nd^ or 3^rd^ trimester. citalopram / escitalopram N=304 (43.5%), sertraline N=118 (16.9%), paroxetine N=92 (13.2%), fluoxetine N=74 (10.6%), fluvoxamine N=3 (0.4%), TCA’s N=38 (5.4%), other AD N=115 (16.5%) (mostly venlafaxine) | *AD exposure:*  *Definition*: SSRI usage during pregnancy  *Ascertainment*: Retrospective self-reported usage in 4-week windows from 2 questionnaires covering conception to week 29+.  *Mental disorder:*  *Definition*: Not defined  *Ascertainment*: Not ascertained | *AD exposure:*  *Definition*: Exposed during the 6-months prior to pregnancy, but not during pregnancy  *Ascertainment:*? as exposed (pre-conception data collection not specifically mentioned)  *Mental disorder:*  *Definition*: depressive symptoms  *Ascertainment*: score of >2 on self-reported Hopkins Symptom Checklist-5 (SCL-5) at week 17 gestation. | *AD exposure*: 1.1%  *Mental disorder:* 6.5% | *Definition*: A birth weight smaller than 2,500g  *Ascertainment*: Medical Birth Registry  *Prevalence*: 2.6% (3.1%) |
| El Marroun et al. (2012) | Prospective cohort (Generation R) | Rotterdam, Netherlands  All pregnant women with delivery date between 04/2002- 01/2006 were eligible (N=9,778 mothers enrolled).  Further excluded those who enrolled after delivery, fetal deaths, multiple births, lost to follow-up, SSRI information unavailable, SSRI only prior to pregnancy; study sample size N=7,696 | SSRIs  Other data NR | *AD exposure:*  *Definition*: SSRI usage during pregnancy  *Ascertainment*: Retrospective self-reported usage asked at each trimester, covering date of conception to third trimester. ‘Large majority’ gave permission to contact pharmacy, 56.4% of women had prescription records. Yules Y = 0.94 for agreement of ascertainment method.  *Mental disorder:*  *Definition*: Not defined  *Ascertainment*: Not ascertained | *AD exposure:*  *Definition*: No SSRI usage during pregnancy  *Ascertainment:*As exposed  *Mental disorder:*  *Definition*: clinically relevant depressive symptoms  *Ascertainment*: score >0.75 on the 6-item depression scale of the self-reported Brief Symptom Inventory at average 20.6 wks gestation.  *Prevalence*: NR | *AD exposure*: 1.3%  *Mental disorder:*NR | *Definition*: A birth weight smaller than 2,500g  *Ascertainment*: extracted from medical records  *Prevalence*: NR (NR) |

*Prevalence in the whole study sample including women without mental health problems, prevalence in brackets for LBW is for analysed sample only (i.e. depressed, exposed group and depressed, unexposed group )

SSRI Selective serotonin reuptake inhibitors, AD antidepressants, LBW low birth weight, NR not reported, TCA tricyclic antidepressant, PS propensity score
