# Supplementary material for: A systematic review of maternal antidepressant use in pregnancy and short- and long-term offspring’s outcomes
Source: Arch Womens Ment Health. 2017 Oct 12;21(2):127–40. doi: 10.1007/s00737-017-0780-3 (PMC5856864; doi:10.1007/s00737-017-0780-3)
Supplement: Supplementary file 2 — (DOCX 23.3 kb) [file 737_2017_780_MOESM2_ESM.docx]

**Table S1b. Analysis and results - low birth weight outcomes**

| **Study** | **No. exposed** | **No. non-exposed** | **Characteristics (between group differences)*** | **Other pharmacology usage during pregnancy (between group differences)*** | **Mental health status (between group differences)***  **Other treatments** | **Analysis findings** and methods** | **Covariates adjusted for in analysis** |
| --- | --- | --- | --- | --- | --- | --- | --- |
| Oberlander et al. (2006) | 1) PS matched  817  2) Non-PS matched  1,451 | 1) PS matched  805  2) Non-PS matched  14,234 | 1) PS matched samples on characteristics.  2) Data for non-PS matched sample:  *Mean age* 29.5 (**exposed**)  *Mean no. prenatal visits* 11.1 (**non-exposed**)  *Mean income decile* 5.3 (NGD)  *Proportion drugs subsidised through welfare programme in year prior to becoming pregnant* 0.09 (**exposed**) | 1) PS matched samples on characteristics  2) For non-PS matched sample users of other AD, benzodiazapines or antipsychotics were excluded | 1) PS matched samples on characteristics.  2) Data for non-PS matched sample:  *Proportion ICD-9 code* 90% exposed, 100% **non-exposed**  *Mean no. visits to psychiatrist in year prior to pregnancy* 0.216 (**exposed**)  *Mean no. of times diagnosed as having depression in year prior pregnancy* 0.842 (**exposed**)  *Mean no. of times receiving 3-digit ICD-9 code indicating depression in year prior to pregnancy* 0.221 (**exposed**)  Mean no. of times provided counselling by GP in previous year 0.491 (**exposed**)  Other treatments NR. | Mean difference in incidence of LBW  1) PS matched samples  0.033 (95% CI 0.007, 0.059, P=0.02)  Unclear how analysed  2) Non-PS matched sample  0.005 (-0.01, 0.02, P=0.51)  2-tailed Fisher exact test | 1) PS matched on  *Prepregnancy*: No. visits pyschiatrist; No. times diagnosed depessed, incidated depressed, other mental health disorder not depression, provided counselling by GP, visits physician; income decile; drugs subsidised  *During pregnancy*: Age; No. prenatal visits; diagnosed depressed, indicated depressed; No. times diagnosed depessed; No. treatments by pyschiatrist; Filled prescription for antipsychotic, tricyclic AD.  2) Non-PS matched sample unadjusted |
| Jensen et al. (2013) | 1,300 | 2,666 | NR (data presented is not in mutually exclusive groups) | NR (data presented is not in mutually exclusive groups) | All women in both groups have diagnoses, no other data on symptoms or service use reported  Other treatments NR. | Compared to women with no diagnosis before end of pregnancy and no AD before or during pregnancy (i.e. not a group included in this review):  Exposed:  No AD prior to pregnancy, AD during pregnancy N=166: aHR 1.44 (0.89, 2.31)  AD prior to pregnancy, AD during pregnancy N=1,134: aHR 1.42 (1.20, 1.68)  Unexposed:  No AD prior to pregnancy, no AD during pregnancy N=740: aHR 0.91 (0.72, 1.16)  AD prior to pregnancy, no AD during pregnancy N=1,926: aHR 1.04 (0.92, 1.20)  Multivariable Poisson regression | Age at delivery, calendar year of delivery, sex of new-born, use of antiepileptics, antipsychotics, other medicine, smoking status, employment status. |
| Nordeng et al. (2012) | 699 | 1,048 | *Proportion maternal age between 20-29* 0.437 (NGD)  *Proportion multiparous* 0.505 (NGD)  *Proportion tertiary education* 0.476 **(non-exposed)**  *Proportion married* 0.899 **(non-exposed)**  *Proportion normal prepregnancy* *BMI* 0.50 **(non-exposed)**  *Proportion not smoking during pregnancy* 0.790 **(non-exposed)**  Proportion folic acid use 0.448 (NGD)  *Proportion hospitalised during pregnancy* (0.105) **(exposed)**  *Proportion asthma* 0.084 (NGD) | *Proportion used other psychotropic drugs* 0.116 (**exposed**)  *Proportion used analgesics* 0.669 (NGD) | *Proportion depressive symptoms*:  week 17, 0.292 **(exposed)**  week 30, 0.286 **(exposed)**  *Proportion lifetime history of depression* 0.876  (NGD)  Other treatments NR. | Compared to women not exposed before and during pregnancy (i.e. not a group included in this review):  Exposed: aOR 0.93 (95% CI 0.55, 1.58)  Unexposed: aOR 0.62 (0.33, 1.16)  Multivariable logistic regression. | Level of depression at week 17, gestational length, sex of child, maternal age at delivery, parity, prepregnancy BMI, CV disease during pregnancy, NSAID use, folic acid use, smoking during pregnancy  Significant confounders retained using manual backward selection. |
| El Marroun et al. (2012) | 99 | 570 | *Mean maternal age* 27.6 years **(exposed)**  *Mean maternal BMI* 25.2 (NGD)  *Mean parity* 0.65 (NGD)  *Proportion higher education* 0.195 **(exposed)**  *Proportion Dutch* 0.274 **(exposed)**  *Proportion no smoking in pregnancy* 0.583 (NGD)  *Proportion no alcohol in pregnancy* 0.558 (NGD)  *Proportion used cannabis in pregnancy* 0.058 (NGD)  *Proportion family income >€2000* 0.284 (**exposed**) | Proportion used benzodiazapines 0.048 **(exposed)** | Mean depression score 1.35 **(non-exposed)**  Mean anxiety score 1.04 (NGD)  Other treatments NR. | Compared to women with low depression score and not exposed (i.e. not a group included in this review):  Exposed: aOR 0.96 (0.65, 1.42) P=0.82  Unexposed: aOR 1.65 (0.77, 3.56) P=0.20  Multivariable logistic regression | Maternal age, BMI, parity, sex of child, education, ethnicity, maternal smoking, drinking and benzodiazapine use, gestational age at birth. |

*Between group differences were either reported in the paper or calculated from summary statistics provided (two-sided alpha=0.05 for t-test, chi-square), group listed (exposed, non-exposed) is the group with the statistically significant higher proportion / mean score for the listed characteristic, NGD indicates No Group Difference on the characteristics; some characteristics and between-group differences in characteristics were calculated using data reported in the paper

** as reported in the paper

BMI body mass index, aOR adjusted odds ratio; aHR adjusted hazard ratio, CI confidence interval, CV Cardiovascular, NSAID Nonsteroidal anti-inflammatory drugs, AD antidepressants
