# Supplementary material for: A systematic review of maternal antidepressant use in pregnancy and short- and long-term offspring’s outcomes
Source: Arch Womens Ment Health. 2017 Oct 12;21(2):127–40. doi: 10.1007/s00737-017-0780-3 (PMC5856864; doi:10.1007/s00737-017-0780-3)
Supplement: Supplementary file 3 — (DOCX 20.3 kb) [file 737_2017_780_MOESM3_ESM.docx]

**Table S2. Quality assessment for LBW outcomes**

|  | Selection /4 | Comparability /2 | | Outcome /2 | Total /8 |
| --- | --- | --- | --- | --- | --- |
|  |  | Controls for depression  severity/1 | Controls for 2 of 3: other drugs, smoking, alcohol /1 |  |  |
| Nordeng et al. (2012) | 3 | 1 | 0 | 1 | 5 |
| Jensen et al. (2013) | 4 | 0 | 1 | 2 | 6 |
| El Marroun et al. (2012) | 3 | 0 | 1 | 1 | 5 |
| Oberlander et al. non-PS (2006) | 4 | 0 | 0 | 2 | 6 |
| Oberlander et al. PS (2006) | 4 | 0 | 0 | 2 | 6 |

PS propensity score matched sample
