# Supplementary material for: A systematic review of maternal antidepressant use in pregnancy and short- and long-term offspring’s outcomes
Source: Arch Womens Ment Health. 2017 Oct 12;21(2):127–40. doi: 10.1007/s00737-017-0780-3 (PMC5856864; doi:10.1007/s00737-017-0780-3)
Supplement: Supplementary file 4 — (DOCX 30.2 kb) [file 737_2017_780_MOESM4_ESM.docx]

**Table S3a: Study characteristics – neurobehavioural and neurodevelopmental outcomes**

|  |  |  |  | **Group definition and ascertainment** | |  |  |
| --- | --- | --- | --- | --- | --- | --- | --- |
| **Study** | **Study design** | **Location and recruitment, exclusions** | **Antidepressants studied, quantity and duration of exposure** | **Exposed group** | **Unexposed group** | **Prevalence*** | **Neurodevelopmental / neurobehavioural outcomes reported*** |
| **Neurobehavioural** | | | | | | | |
| Suri et al. (2011) | Prospective cohort | University of California Institute for Neuroscience and Human Behaviour, USA. 90 of the 93 women who completed a previous study on AD during pregnancy (Suri et. al. 2007) were invited to participate. In the original study women with major depressive disorder (MDD) were recruited from local clinics, direct referral and self-referral in the 1^st^ trimester. Exclusions: (original recruitment), women who were not between 18-45, were actively suicidal, met DSM-IV for another current Axis 1 disorder, positive urine drug screen, used medications with documented adverse effects on fetus (not listed). Controls were recruited from outpatient obstetric clinics and self-referral. Exclusions as for cases, but without current SSM-IV axis I disorder. A further N=4 were excluded for no child outcomes at 1 week, N=5 at 6-8 wks. Sample size N=64. | NR, mentioned the majority of exposures were to sertraline and fluoxetine.  Mean daily doses of sertraline: 90.5 mg (SD 50.3), fluoxetine 22.5 (7.5) | *AD exposure:*  *Definition*: Took AD for more than 50% of pregnancy  *Ascertainment*: Self-reported monthly visit with a study physician who collected information about medications and dosages  *Mental disorder:*  *Definition*: Depressive episodes  *Mental disorder criteria*: DSM-IV lifetime diagnosis of MDD using SCID administered by a study physician on study entry (Suri et. al. 2007) during pregnancy | *AD exposure:*  *Definition*: Elected not to take AD during pregnancy, discontinued AD in the 1^st^ trimester and/or had AD exposure <10 days  *Ascertainment*: As exposed  *Mental disorder:*  *Definition*: As exposed    *Ascertainment*: As exposed | N/A – very few non-exposed, non-depressed mothers were recruited, indicating selection in a case-control design | (1) Neonate behaviour  *Definition*: not defined (mean scores)  *Ascertainment*: Brazelton Neonatal Behavioural Assessment Scale (BNBAS) summary scores for a. Habituation, b. Orientation, c. Motor, d. Defense, e. Range of state, f. Regulation of state, g. Autonomic stability, h. Reflexes cluster groups, administered 1, and 6-8 wks, after delivery by 1 of 2 trained blinded raters. |
| **Neurodevelopmental** | | | | | | | |
| El Marroun et al. (2014) | Population based regional prospective cohort (Generation R) | All pregnant women in Rotterdam between 04/02 & 01/06, N=8,880 mothers. Excluded N=650 missing information on SSRI’s, N=782 children without pre and postnatal follow-up, N=146 due to use of SSRIs before pregnancy; study sample size N=7,302 children.  A further N=1,326 were excluded from the analysis because child outcomes were not available | SSRIs.  Out of 69 exposed, N=35 (50.7%) in 1^st^ trimester only, N=34 (49.3%) in 1^st^ & other trimesters. N=38 women used paroxetine, N=17 fluoxetine, N=11 sertraline, N=6 fluvoxamine, N=4 citalopram. Some of these used multiple SSRIs. Mean duration of SSRI use was 257 days. | *AD exposure:*  *Definition*: Any use during pregnancy  *Ascertainment*: (1) SR questionnaire covering previous trimester, type of medication taken and when it was used. SSRI use assessed from this. (2) Contact of pharmacy for consenting women (“large majority” consented), due to delays in linkage only 60.2% of records were retrieved.  Yules Y 0.93 for agreement for exposure from both methods  *Mental disorder:*  None, exposed group defined on AD use only | *AD exposure:*  *Definition*: No maternal SSRI use  *Ascertainment:*  As for exposed group  *Mental disorder:*  *Definition*: Depressive symptoms during pregnancy  *Ascertainment*: A score >0.75 on the 6-item depression scale of the Brief Symptom Inventory (BSI) at av. 20.6 wks gestation was used to indicate clinically relevant depressive symptoms | *AD exposure*: 1.17%  *Mental disorder:* 14% (whole period) | (1) Pervasive developmental problems  *Definition*: Score of ≥7 on pervasive developmental problems subscale of the Child Behaviour Checklist age 1.5-5 (CBCL 1.5-5)  *Ascertainment*: Mother reported, at 18 mo, age 3 (also father reported at age 3) and age 6  (2) Autistic symptoms  *Definition*: Not defined (mean scores)  *Ascertainment*: Mother reported Social responsiveness scale (SRS) at age 6  (3) Specific autistic symptoms  *Definition*: Not defined (mean scores)  *Ascertainment*: Mother reported a. Cognition, b. Social Communication and c. Autistic Mannerism subscales on the SRS at age 6 |
| Pedersen et al. (2013) | Prospective cohort (Danish National Birth Cohort) | Pregnant women in Denmark recruited by primary care practitioners from 1996-2002 (50% practitioners nationwide took part), with 60% enrolment rate (N=101 042 pregnancies).  Excluded those who did not complete both prenatal interviews (leaving 82 687 women), those taking psychotropic medications other than antidepressants (N=741), women with schizophrenia, drug addictions or unknown psychiatric diagnosis (N=792), women with multiple births, fetal deaths, women who gave birth on or before Nov 30, 2001 (leaving 34 661 women). A random sample of 1,109 non-exposed, non-depressed controls were selected. Further excluded N=95 children participating in another study, and children whose mothers had died, refused further contact, emigrated or had a protected address. Total sample size N=1406  A further 458 were excluded from the analysis because child outcomes were not available. Total sample size N=948 | Any drug under WHO-defined Anatomic Therapeutic Chemical Classification System (ATC) group N06A  Including women without child outcomes (N=212), Monotherapy: N=50 (23.6%) fluoxetine, N=37 (17.5%) citalopram, N=38 (17.9%) paroxetine, N=47 (22.2%) sertraline, N=10 (4.7%) TCA, N=13 (6.1%) other (e.g. venlafaxine). Combination therapy N=10 (4.7%) >1 SSRI, N=7 (3.3) other AD with either SSRI or TCA. | *AD exposure:*  *Definition*: Any use during pregnancy  *Ascertainment*: Self-reported use  *Mental disorder:* NR whether mental disorder questions were also applied to define exposed group | *AD exposure:*  *Definition*: No use during pregnancy.  *Ascertainment:* Self-reported use  *Mental disorder:*  *Definition*: Unclear - NR how responses to questions were used to classify women with depression.  *Ascertainment*: Four questions were asked at 17 wks gestation (1) ever suffered (2) did you see a doctor or psychologist (3) name of disorder (4) experienced symptoms of it during pregnancy, and at 32 wks gestation asked if they had experienced a psychiatric disease during pregnancy, and which. | N/A - non-exposed, non-depressed mothers were selected in a case-control design | (1) Abnormal behaviour  *Definition*: Abnormal score on the Strengths and Difficulties Questionnaire (SDQ) (a. Total Difficulties, b. Emotional, c. Hyperactivity, d. Conduct, e. Peer, f. Prosocial subscales) as derived from normative data  *Ascertainment*: Parent reported SDQ at age 4-5  (2) Behaviour:  *Definition*: Not defined (mean score)  *Ascertainment*: Parent reported SDQ (a. Total Difficulties, b. Emotional, c. Hyperactivity, d. Conduct, e. Peer, f. Prosocial subscales) at age 4-5 |
| Nulman et al. (2012) | Prospective cohort | Recruited from pregnant women who sought counselling at the Hospital for Sick Children, Toronto, on the pregnancy safety of medications, N=608 women contacted the program. Excluded mothers taking polytherapy for depression, known teratogens, substance abuse, psychiatric conditions other than depression (if depressed), children of gestational age <37 wks, mothers or children with cognitive problems unrelated to in utero exposure, or inadequate English proficiency. A non-exposed, non-depressed group were selected as the first women who contacted programme (unclear how N was decided). Excluded N=381.  A further 49 could not be contacted for the follow-up. Total sample size N=178. | Venlafaxine and SSRIs (in this sample, sertraline, paroxetine, citalopram, fluoxetine, fluvoxamine)  Standardised daily dose: venlafaxine range 0.25 to 3.75, SSRI 0.40 to 4.00. 81 (65.3%) of exposures throughout pregnancy, N=21 (16.9%) in 1^st^ trimester only, N=4 (3.2%) in 1^st^ & 2^nd^, N=2 (1.6%) in 2^nd^ only, N=11 (8.9%) in 2^nd^ and 3^rd^, N=5 (4.0%) in 3^rd^ only. | *AD exposure:*  *Definition*: Taking antidepressants during pregnancy. Exposed-1 = venlafaxine, Exposed-2=SSRI  *Ascertainment*: Retrospective self-reported usage  *Mental disorder:*  *Definition*: Depressive episodes  *Ascertainment*: According to DSM-IV diagnosed by the woman’s psychiatrist | *AD exposure:*  *Definition*:  Discontinued antidepressants prior to pregnancy  *Ascertainment:*  As exposed  *Mental disorder:*  *Definition*: Depressive episodes  *Ascertainment*: As exposed | N/A - non-exposed, non-depressed mothers were selected in a case-control design | (1) IQ  *Definition*: not defined (mean scores)  *Ascertainment*: Weschsler Preschool and Primary Scale of Intelligence 3rd Edition (a. Full Scale, b. Verbal Scale & c. Performance Scale) administered by a psychometrist  (2) Problem behaviour  *Definition*: Score ≥64 on a. Total problems, b. Internalising, c. Externalising scales of CBCL  *Ascertainment*: mother reported at age 3-6 yrs 11 mo  (3) Behaviour  *Definition*: not defined (mean scores)  *Ascertainment*: mother reported CBCL, (a. Total problems) at age 3-6 yrs 11 mo  (4) ADHD & comorbid disorders  *Definition*: Score ≥65 on a. Total problems, and b. DSM total symptoms of Connors’ Parent Rating Scale  *Ascertainment*: Mother reported at age 3-6 yrs 11 mo  (5) ADHD & comorbid scale  *Definition*: Not defined (mean score)  *Ascertainment*: Mother reported a. Total problems, and b. DSM total symptoms of Connors’ Parent Rating Scale at age 3-6 yrs 11 mo |
| Pederson et al. (2010) | Prospective cohort (Danish National Birth Cohort) | Pregnant women recruited by primary care practitioners from 1996-2002 (50% practitioners nationwide took part), with 60% enrolment rate (N=101 042 pregnancies). Excluded those without both prenatal interviews (leaving 82 687 women), those taking psychotropic medications other than antidepressants (N=741). Sample size 81 946 women. Further excluded multiple births N=1,853 and N=228 without child outcome data. | Any drug under WHO-defined Anatomic Therapeutic Chemical Classification System (ATC) group N06A.  NR. Results stratified by 1^st^ trimester exposure only, and 2^nd^/3^rd^ trimester exposure but N not reported. Some results also stratified by type of AD (SSRI, TCA) | *AD exposure:*  *Definition*: Any use during pregnancy  *Ascertainment*: Self-reported use  *Mental disorder:* NR whether mental disorder questions were also applied to define exposed group | *AD exposure:*  *Definition*: No use during pregnancy.  *Ascertainment:* Self-reported use  *Mental disorder:*  *Definition*: Unclear - NR how responses to questions were used to classify women with depression.  *Ascertainment*: Four questions were asked at 17 wks gestation (1) ever suffered (2) did you see a doctor or psychologist (3) name of disorder (4) experienced symptoms of it during pregnancy, and at 32 wks gestation asked if they had experienced a psychiatric disease during pregnancy, and which. | *AD exposure*: 0.51%  *Mental disorder:* 1.1% (assuming all exposed depressed) | (1) *Definition*: Meets developmental milestones  *Ascertainment*: Mother reported at 6 and 19 mo. At 19 mo: a. Gross-motor – going up stairs with support, b. Age at which child walked without support, c. Fine motor – taking off socks and shoes when asked to, d. Fine motor – drinking from ordinary cup without help, e. Attention – being occupied alone for ≥15 min, f. Cognition –Bringing things when told to, g. Cognition – making marks on table or paper, h. Cognition – aligning picture correctly, i. Language – using word-like sounds to tell what s/he wants, j. Language – mentioning >25 names of different things, k. Language – Using 2-word sentences, l. Failed ≥1 milestone |
| Santucci et al. (2014) | Prospective cohort | Recruited at or before 20 wks gestation from 2 sites, Cleveland and Pittsburgh (USA), via self-referral, physician referral, advertising and screening in obstetrical suites (Wisner et. al. 2009). N=272. Exclusions were psychosis, bipolar disorder, substance use, exposure to benzodiazepines or any US FDA pregnancy class D or X drugs, multiple births or major medical disorders, no delivery data (N=137).  Study sample size N=236.  Excluded N=71 for no child outcome data. Sample size N=165. | SSRIs and venlafaxine  Out of 41 exposed, N=29 (71%) exposed continuously during pregnancy. N=6 (14.5%) in 1^st^ and/or 2^nd^ trimester but not 3^rd^, N=6 (14.5%) not 1^st^.  N=15 sertraline, N=11 fluoxetine, N=7 escitalopram, N=5 citalopram, N=1fluvoxamine, N=1 paroxetine, N=5 venlafaxine | *AD exposure:*  *Definition*: Any exposure during pregnancy  *Ascertainment*: Self-reported (?) part (?) retrospective, each week from conception (Wisner et. al. 2009)  *Mental disorder criteria*:  *Definition*: Major depressive disorder (MDD) at some point in the pregnancy  *Ascertainment*: SCID for DSM-IV for MDD at 20, 30 and 36 wks gestation. | *AD exposure:*  *Definition*: No use during pregnancy.  *Ascertainment:* As exposed  *Mental disorder criteria*:  *Definition*: As exposed  *Ascertainment*: As exposed | *AD exposure*: N/A, number of controls may have been fixed  *Mental disorder:* N/A, number of controls may have been fixed | (1) *Definition*: Normal child behavioural development  *Ascertainment*: ≥75 percentile on total score and subscales of the Behavioural Rating Scale (BRS) of the Bayley Scales of Infant Development (BSID-II) a. Total score, b. Attention/arousal (12 wk only), c. Orientation / engagement, d. Emotional regulation, e. Motor quality at 26, 52, 78 wks. NR how administered however BSID-II require independent trained observers.  (2) Child mental development  *Definition*: not defined (mean scores)  *Ascertainment*: Age-adjusted standardised scores on the Mental Development Index (MDI) of the BSID-II at 12, 26, 52, 78 wks  (3) Child psychomotor development  *Definition*: not defined (mean scores)  *Ascertainment*: Age-adjusted standardised scores on the Psychomotor Development Index (PDI) of the BSID-II at 12, 26, 52, 78 wks |
| Casper et al. (2003) | Prospective cohort | USA. Recruited from those in treatment in a Women’s Wellness Clinic or other clinicians and who met DSM-IV for major depressive disorder (MDD) during pregnancy. Recruited before or during pregnancy (71%) or after delivery (29%). Reported recruiting N=44. | SSRIs  Of the 31 exposed, 48% took sertraline, 23% fluoxetine, 26% paroxetine, and 3.2% fluvoxamine. Mean average daily doses were sertraline (113.2mg, SD 72.3), fluoxetine (20.0, 11.9), paroxetine (17.2, 10.1) and fluvoxamine (50 mg/d). 45% took throughout whole pregnancy, 71% during 1^st^ trimester, 74% during 3^rd^. | *AD exposure:*  *Definition*: Taking SSRIs at referral (unclear whether for the study, or for treatment) or started SSRI during pregnancy  *Ascertainment*: Self-reported, retrospective dose and timing  *Mental disorder criteria*: MDD  *Definition*:  *Ascertainment*: Interview with SCID DSM-IV Axis I Disorders (assumed at recruitment)  Women also received psychotherapy during pregnancy. | *AD exposure*: Did not take SSRIs during pregnancy  *Definition*: As exposed  *Ascertainment*: As exposed  *Mental disorder:* MDD  *Definition*: As exposed  *Ascertainment*: As exposed  Women also received psychotherapy during pregnancy. | *AD exposure*: NA, no controls  *Mental disorder:* NA, no controls | (1) Child behavioural development  *Definition*: not defined (mean scores)  *Ascertainment*: Total score and subscales of the Behavioural Rating Scale (BRS) of the Bayley Scales of Infant Development (BSID-II) a. Total score, b. Attention/arousal, c. Orientation / engagement, d. Emotional regulation, e. Motor quality between 6 and 40 mo. Assessed by one of two clinical child psychologists certified in use of the BSID-II.  (2) Motor quality items  *Definition*: not defined (mean scores)  *Ascertainment*: Items comprising the motor quality factor scale of the (BSID-II) a. Gross motor movement, b. Fine motor movement, c. Control of movement, d. Tremulousness, e. Slow and delayed movement, f. Frenetic movement, g. Hypertonicity, h. Hypotonicity, between 6 and 40 mo. Assessed as above.  (3) Child mental development  *Definition*: not defined (mean scores)  *Ascertainment*: Mental Development Index (MDI) of the BSID-II between 6 and 40 mo. Assessed as above.  (4) Child psychomotor development  *Definition*: not defined (mean scores)  *Ascertainment*: Psychomotor Development Index (PDI) of the BSID-II between 6 and 40 mo. Assessed as above. |

*prevalence in the whole study sample including women without mental health problems, prevalence in brackets for neurodevelopmental outcomes is for analysed sample (i.e. women with mental health problems);

SSRI Selective serotonin reuptake inhibitors, AD antidepressants, LBW low birth weight, NR not reported, TCA tricyclic antidepressant, PS propensity score; SCID Structured Clinical Interview for DSM.
