# Supplementary material for: A systematic review of maternal antidepressant use in pregnancy and short- and long-term offspring’s outcomes
Source: Arch Womens Ment Health. 2017 Oct 12;21(2):127–40. doi: 10.1007/s00737-017-0780-3 (PMC5856864; doi:10.1007/s00737-017-0780-3)
Supplement: Supplementary file 5 — (DOCX 34.1 kb) [file 737_2017_780_MOESM5_ESM.docx]

**Table S3b. Analysis and results - neurodevelopmental and neurobehavioural outcomes**

| **Study** | **No. exposed** | **No. non-exposed** | **Characteristics (between group differences)*** | **Other pharmacology usage during pregnancy (between group differences)*** | **Mental health status (between group differences)***  **Other treatments** | **Analysis findings** and methods** | **Covariates adjusted for in analysis** |
| --- | --- | --- | --- | --- | --- | --- | --- |
| **Neurobehavioural** | | | | | | | |
| Suri et al. (2011) | N=31 | N=14 at wk 1  N=13 at wks 6-8 | *Mean age* 33.7 yrs (NGD)  *Mean education* 17.3 (NGD)  *Mean parity* (NGD)  *Mean gestational age*, wk 38.5 (**exposed shorter**)  *Proportion pre-term births* <37 wk 0.082 (NGD)  *Mean birthweight* 3.3 kg (NGD)  *Mean APGAR 1 min* 7.9 (NGD)  *Mean APGAR 5 mins* 8.8 (NGD)  *Proportion special care admissions* 0.163 (NGD)  *Proportion male* 0.531 (NGD)  Noted that cigarettes and alcohol use were uncommon, numbers NR | Excluded users of medications with documented adverse effects on fetus (not listed). | Hamilton Depression Rating Scale (HDRS) and SCID-IV mood module assessed monthly  Mean average HDRS score, 10.3 (NGD)  Mean maximum HDRS during pregnancy, 17.8 (NGD)  Mean HDRS 8 wks postpartum 7.6 (NGD)  Other treatments NR. | Estimates are mean (SD) F, [df], P  ***(1) BNBAS scores at 6-8 wks***  *a. Habituation* Exposed 6.04 (2.12), Unexposed 4.50 (1.22) 2.16 [2,11] P=0.16  *b. Orientation* Exposed 6.17 (2.13), Unexposed 6.87 (0.85) 1.17 [2,54] P=0.32  *c. Motor* Exposed 5.89 (0.71), Unexposed 6.20 (0.74) 0.97 [2,54] P=0.39  *d. Defense* Exposed 7.19 (0.91), Unexposed 7.00 (1.16) 3.39 [2,54] P=0.04 (NS after Bonferroni correction)  *e. Range of state* Exposed 3.14 (0.96), Unexposed 3.25 (1.04) 0.38 [2,54] P=0.68  *f. Regulation of state* Exposed 4.46 (1.05), Unexposed 4.29 (0.95) 0.41 [2,54] P=0.67  *g. Autonomic stability* Exposed 7.48 (0.75), Unexposed 7.67 (0.61) 0.31 [2,54] P=0.74  *h. Reflexes* Exposed 3.13 (2.45), Unexposed 2.46 (1.61) 1.65 [2,54] P=0.20  Analysis of covariance | Gestational age at delivery  Models were also adjusted for maximum HDRS during pregnancy, HDRS scores 4 and 8 wks but detailed results were not reported |
| **Neurodevelopmental** | | | | | | | |
| El Marroun et al. (2014) | N=69 for pervasive developmental problems outcome  N=50 specific autistic symptoms outcome | N=376, pervasive developmental problems outcome  N=222 specific autistic symptoms outcome | *Mean age at recruitment* 28.8 (**exposed**)  *Proportion Higher Education* 0.260 (**exposed**)  *Proportion Dutch* 0.324 (**exposed**)  *Proportion never smoked in pregnancy* 0.580 (NGD)  *Proportion ever drank in pregnancy* 0.557 (**non-exposed**)  *Proportion boys* 0.507 (**non-exposed**)  *Mean child birth weight* 3333 g (NGD)  *Mean gestational age at birth wk* 39.7 (NGD) | *Proportion used benzodiazepines* 0.056  (**exposed**) | *Mean BSI scores prenatal* 1.27  (**non-exposed**)  *Mean BSI scores postnatal* 0.46 (**non-exposed**)  Other treatments NR. | Multiple imputation reported as main results for the 4.9% missing data  All compared to non-depressed, non-exposed controls.  ***(1) Pervasive developmental problems***  Exposed: aOR 1.91 (1.13, 3.47) P=0.03  Unexposed: aOR 1.44 (1.07, 1.93) P=0.02  Multivariable generalised estimating equation (GEE), combining ratings at 1.5, 3 and 6 years  ***(2) Autistic symptoms***  Exposed: β 0.15 (0.08, 0.22) P<0.001  Unexposed: β 0.05 (0.01, 0.08) P=0.01  Multivariable linear regression  ***(3) Specific autistic symptoms***  *a. Social cognition*  Exposed: β 0.13 (0.03, 0.23) P=0.01  Unexposed: β 0.05 (-0.01, 0.04) P=0.07  *b. Social communication*  Exposed: β 0.17 (0.09, 0.25) P<0.001  Unexposed: β 0.05 (0.01, 0.09) P=0.02  *c. Autistic mannerism*  Exposed: β 0.12 (0.05, 0.20) P=0.006  Unexposed: β 0.04 (-0.01, 0.07) P=0.07  Multivariable linear regression | Maternal age, education, ethnicity, smoking habits, postnatal depressive symptoms at age 3 years, child sex, gestational age.  Covariates selected on change in estimate method. |
| Pedersen et al. (2013) | N=127 | N=98 | *Mean age* NR (data incompletely reported)  *Proportion nulliparous* 0.533 (NGD)  *Mean BMI* NR (data incompletely reported)  *Proportion prenatal smoking* 0.473 (NGD)  *Proportion > 10 cigarettes /day prenatally* 0.240 (NGD)  *Proportion prenatal alcohol* 0.404 (NGD)  *Mean average alcoholic drinks* (NGD)  *Proportion prenatal caffeine* 0.422 (NGD)  *Mean average cups per day prenatally* NR (data incompletely reported)  *‘High’ is highest level of education proportion* 0.640 (NGD)  *Living with a partner proportion* 0.956 (NGD) | Women taking psychotropic medications other than antidepressants during pregnancy were excluded | Self-reported mood symptoms at 32 wks gestation (Note only one category (‘a lot’) is listed here for brevity, statistical significance was calculated using all categories)  *Proportion felt down or sad* (a lot) 0.293 (**non-exposed**)  *Proportion felt that the future was hopeless* (a lot) 0.182 (**non-exposed**)  *Proportion felt scared or anxious without reason* (a lot) 0.142 (NGD)  *Proportion felt under constant pressure* (a lot) 0.111  (**non-exposed**)  *Proportion DSM-IV current major depression according to self-reported Major Depression Inventory* 0.062 (NGD)  *Proportion self-reported psychiatric disease since delivery* 0.404 (NGD)  *Proportion medical treatment since delivery* 0.290 (**exposed**)  *Proportion untreated disorder since delivery* 0.493 (NGD)  Other treatments NR. | Estimates are for exposed compared to unexposed (95% CI)  ***(1) Abnormal total difficulties and subscale scores***  *a. Total difficulties* aOR 1.3 (0.3, 6.0)  *b. Emotional* aOR 1.6 (0.8, 8.9)  *c. Conduct* aOR 0.6 (0.3, 1.3)  *d. Hyperactivity* aOR 1.8 (0.6, 5.6)  *e. Peer* aOR 0.9 (0.2, 4.8)  *f. Prosocial* aOR 0.5 (0.2, 1.7)  Multivariable logistic regression  ***(2) Total difficulties and subscale scores***  *a. Total difficulties* β -0.7 (-1.8, 0.4)  *b. Emotional* β -0.3 (-0.7, 0.1)  *c. Conduct* β -0.1 (-0.5, 0.3)  *d. Hyperactivity* β -0.2 (-0.7, 0.4)  *e. Peer* β -0.1 (-0.4, 0.2)  *f. Prosocial* β 0.1 (-0.4, 0.5)  Multivariable linear regression | Maternal age, child gender (NR), smoking, alcohol, combined social class (NR). Postnatal maternal depression was adjusted for in secondary analysis but results were not reported. Variables included were guided by DAGs. |
| Nulman et al. (2012) | Exposed-1 N=62 (venlafaxine)    Exposed-2 N=62 (SSRI) | N=54 | *Mean age at delivery* 32.5 yrs (NGD)  *Mean maternal weight gain* 16.3 kg (NGD)  *Mean maternal IQ* 109 (NGD)  *Proportion cigarette use* 0.079 (NGD)  *Proportion alcohol use* 0.022 (NGD)  *Proportion household income ≥ $50k* (NGD)  *Proportion Hollingshead-rated socio-economic status ≥ medium* 0.933 (NGD)  *Mean gestational age* 39.2 wks (NGD)  *Mean birth weight* 3457 g (NGD)  *Mean percentile child’s height* 57.1 (NGD)  *Mean percentile child’s weight* 62.2 (NGD)  *Mean percentile child’s head circumference* 58.6 (NGD)  *Mean age at testing* 49.6 mo  (**non-exposed**)  *Proportion male* 0.539 (NGD)  Overall estimates slightly inaccurate due to missing data | Women taking known teratogens and polytherapy for depression were excluded  Other usage NR | All women had DSM-IV depressive episode  Mean duration of depression 10.6 yrs (NGD)  Mean duration of pharmacotherapy 8.2 yrs (**exposed-1 longer than non-exposed**)  Mean severity of depression during pregnancy (VAS) 3.47 (**both non-exposed and exposed-2 worse than exposed-1**)  Mean severity of depression at time of child testing (CES-D z score) 0.37 (NGD)  Unexposed group were untreated. | Non-exposed controls could not be seperated out in the analysis for the following;  ***(1) IQ, (3) Behaviour, (5) ADHD & comorbid scale***  Estimates are unadjusted proportions (significance)  ***(2) Problem behaviour***  *a. Total score* Exposed-1 0.100, Exposed-2 0.115, Unexposed 0.070 (NS)  *b. Internalising* Exposed-1 0.068, Exposed-2 0.095, Unexposed 0.040 (NS)  *c. Externalising* Exposed-1 0.082, Exposed-2 0.080, Unexposed 0.090 (NS)  ***(4) ADHD & comorbid disorders***  *a. Total problems* Exposed-1 0.080, Exposed-2 0.170, Unexposed 0.020 (P=0.03)  *b. DSM total symptoms* Exposed-1 0.120, Exposed-2 0.120, Unexposed 0.130 (NS)  χ^2^ (non-depressed, non-exposed controls included) | Only outcomes 1, 3 and 5 reported adjusted. |
| Pederson et al. (2010) | N=313 at 6 months N=294 at 19 months | N=363 at 6 months  N=310 at 19 months | *Mean age* NR (data incompletely reported) (**exposed**)  *Proportion nulligravida* 0.303 (NGD)  *Proportion nulliparous* 0.482 (NGD)  *Proportion no previous spontaneous abortion* 0.769 (NGD)  *Mean BMI kg/m^2^* NR (data incompletely reported) (NGD)  *Proportion prenatal smoking* 0.473 (NGD)  *Proportion >10 cigarettes /day prenatally* 0.296 (NGD)  *Proportion prenatal alcohol* 0.406 (NGD)  *Mean alcoholic drinks per day prenatally* NR (data incompletely reported) (NGD)  *Proportion prenatal coffee* 0.473 (NGD)  *Mean cups of coffee per day prenatally* NR (data incompletely reported) (NGD)  *Proportion ‘High’ is highest level of education* 0.558 (**exposed**)  *Proportion living with a partner* 0.945 (NGD)  Data are presented in the paper includes those excluded in the analysis for missing outcome data | Excluded women who took psychotropic medications other than antidepressants | Note only one of categories (a lot) is listed here, significance was reported for all data.  Self-reported mood symptoms at 32 wks gestation  Proportion felt down or sad (a lot) 0.327 (non-exposed worse)  Proportion felt that the future was hopeless (a lot) 0.167 (**non-exposed**)  Proportion felt scared or anxious without reason (a lot) 0.189 (**non-exposed**)  Proportion felt under constant pressure (a lot) 0.113 (**non-exposed**)  Self-reported mood symptoms at 6 months after birth  Proportion felt down or sad (a lot) 0.164 (**non-exposed**)  Proportion felt that the future was hopeless (a lot) 0.101 (**non-exposed**)  Proportion felt scared or anxious without reason (a lot) 0.142 (NGD)  Proportion felt under constant pressure (a lot) 0.129 (**non-exposed**)  Data presented in the paper includes those excluded in the analysis for missing outcome data  Other treatments NR. | Estimates are for any AD, with any exposure, exposed compared to unexposed  ***(1) Meets developmental milestones***  *a. Gross-motor – going up stairs with support* aOR 1.0 (0.50, 2.05)  *c. Fine motor – taking off socks and shoes when asked to* aOR 1.1 (0.68, 1.64)  *d. Fine motor – drinking from ordinary cup without help* aOR 3.4 (0.66, 17.0)  *e. Attention – being occupied alone for ≥15 min* aOR 1.2 (0.72, 1.87)  *f. Cognition –Bringing things when told to* aOR 0.8 (0.28, 2.45)  *g. Cognition – making marks on table or paper* aOR 1.3 (0.59, 3.07)  *h.* *Cognition – aligning picture correctly* aOR 1.0 (0.68, 1.42)  *i.* *Language – using word-like sounds to tell what s/he wants* aOR 1.4 (0.59, 3.11)  *j.* *Language – mentioning >25 names of different things* aOR 1.7 (0.95, 3.10)  *k.* *Language – Using 2-word sentences* aOR 1.2 (0.83, 1.74)  *l.* *Failed ≥1 milestone* aOR 2.1 (0.95, 4.86)  Multivariable logistic regression  *b. Age at which child walked without support* difference (days) 13.6 (4.0, 23.3)  Multivariable linear regression | Maternal age, sex of child (NR), age at interview, breastfeeding (not defined, NR), problems during pregnancy (not defined, NR), mother-child connection (not defined, NR), postnatal symptoms of depression and postnatal difficulties (not defined, NR).  Variables included guided by DAGs.  Results 1a, c-l also stratified by exposure window, 1b by exposure window and type of AD (AD, SSRI, TCA). Unspecified analyses were stratified by maternal smoking and drinking during pregnancy. |
| Santucci et al. (2014) | N=41 | N=27 | *Mean age* 30.3 yrs (NGD after BF correction)  *Proportion White* 0.765 (**exposed**)  *Proportion completed university* 0.588 (**exposed**)  *Proportion employed* 0.456 (NGD)  *Proportion married / cohabiting* 0.721 (**exposed**)  *Proportion prepregnancy BMI ≥30* 0.338 (NGD)  Proportion parity ≥3 0.338 (NGD)  *Proportion smoked during pregnancy* 0.118 (NGD)  *Proportion drank during pregnancy* 0.309 (NGD)  *Proportion gestational age <37 wks* 0.176 (NGD)  *Proportion female* 0.559 (NGD after BF correction)  *Mean birth weight* 3,333g (NGD)  *Mean length* 50.5cm (NGD)  *Mean head circumference* 34.3cm (NGD)  *Proportion ever breastfed* 0.662 (NGD) | Excluded women who took benzodiazepines or any US FDA pregnancy class D or X drugs | Mean SIGH-ADS at 20 wks mean 15.7 (NGD after Bonferroni correction)  Proportion lifetime anxiety (self-reported) 41.2% (NGD)  Other treatments NR. | Estimates are percentages (SE)  ***(1) Normal child behavioural development***  *a. Total score at 78 wks* Exposed 72% (9.2), Unexposed 81.8 (12.2)  *b. Attention/arousal at 12 wks* Exposed 81.8 (6.8), Unexposed 72.2 (10.9)  *c. Orientation / engagement at 78 wks* Exposed 76.9 (8.4), Unexposed 90.9 (9.1)  *d. Emotional regulation at 78 wks* Exposed 65.4 (9.5), Unexposed 72.7 (14.1)  *e. Motor quality at 78 wks* Exposed 53.8 (10.0), Unexposed 72.7 (14.1)  Estimates are mean (SE)  ***(2) Child mental development at 78 wks***  Exposed 98.1 (2.9), Unexposed 100.5 (4.8)  ***(3) Child psychomotor development at 78 wks*** Exposed 99.3 (1.7), Unexposed 105.2 (3.1)  Also reported estimates of unadjusted repeated measures mixed models (& logistic models) with a random intercept and unstructured covariance matrix, but these models also included the non-depressed, non-exposed group. | Unadjusted |
| Casper et al. (2003) | N=31 | exposed N=13 | *Proportion married* 0.89 (NGD)  *Proportion miscarriages* 0.36 (NGD)  *Proportion alcohol use* (<9 drinks in pregnancy=0)  (NGD)  *Proportion tobacco use* 0  (NGD)  *Proportion illicit drug use* 0  (NGD)  *Proportion prenatal vitamins* 0.89 (NGD)  *Proportion vegetarian diet* 0.07 (NGD)  *Proportion illness or flu in pregnancy* 0.32 (NGD)  *Proportion exercise* 0.73 (NGD)  *Proportion caesarean* 0.27 (NGD)  *Mean age* 35.4 yrs (NGD)  *Mean education* 16.9 yrs  (NGD)  *Mean parity* 1.6 (NGD)  *Mean hours in labour* 9.3 (NGD)  *Mean weight gain in pregnancy* 30.2 lb (NGD)  *Proportion preterm* 0.05 (NGD)  *Proportion first born* 0.45 (NGD)  *Proportion admission to NICU* 0.16 (NGD)  *Proportion breast feeding* (not defined) 0.89 (NGD)  *Proportion SSRI while breast feeding* 0.45 (**exposed**)  *Mean gestational age* wk 39 (NGD)  *Mean birth weight* 3384 g  (NGD)  *Mean birth length* 50.1 cm  (NGD)  *Mean APGAR* 1 min 7.4 (**exposed lower APGAR**)  *Mean APGAR* 5 min 8.6 (**exposed lower APGAR**)  *Mean age at follow up* 14.3 mo (NGD)  *Proportion weight* 47.9 (NGD)  *Proportion height* 44.2 (NGD)  *Proportion fronto-occipital circumference* 53.0 (NGD) | NR | Depression ratings on Likert scale  Mean 1^st^ trimester 4.7 (NGD)  Mean 2^nd^ trimester 5.1 (NGD)  Mean 3^rd^ trimester 5.2 (NGD)  Mean BDI maximum score mean 22.1 (NGD)  Women in both groups received psychotherapy. | Estimates are unadjusted mean (SD), adjusted [F] P value  ***(1) Child behavioural development***  *a. Total score* Exposed 76.0 (24.6) Unexposed 89.5 (15.4), [2.57], P=0.12  *b. Attention/arousal* Exposed 76.6 (25.6) Unexposed 94.0 (7.1), [1.2], P=0.31  *c. Orientation / engagement* Exposed 73.0 (27.2) Unexposed 76.6 (30.1), [0.02], P=0.88  *d. Emotional regulation* Exposed 78.3 (27.6) Unexposed 87.5 (25.3), [0.07], P=0.79  *e. Motor quality* Exposed 68.6 (29.0) Unexposed 88.8 (20.2), [4.02], P=0.05  ***(2) Child motor development***  *a. Gross motor movement* Exposed 4.43 (0.68) Unexposed 4.77 (0.44), [2.01], P=0.17  *b. Fine motor movement* Exposed 4.71 (0.46) Unexposed 5.0 (0), [2.22], P=0.15  *c. Control of movement* Exposed 4.60 (0.56) Unexposed 4.77 (0.44), [0.55], P=0.46  *d. Tremulousness* Exposed 4.87 (0.34) Unexposed 5.00 (0), [3.37], P=0.08  *e. Slow and delayed movement* Exposed 4.83 (0.38) Unexposed 4.92 (0.28), [0.06], P=0.81  *f. Frenetic movement* Exposed 4.87 (0.43) Unexposed 5.00 (0), [2.14], P=0.15  *g. Hypertonicity* Exposed 4.97 (0.18) Unexposed 5.00 (0), [0.74], P=0.40  *h. Hypotonicity* Exposed 4.90 (0.31) 4.92 0.28), [0.05], P=0.83  **(*3) Child mental development*** Exposed 91.0 (13.3) Unexposed 94.3 (7.5), [2.12], P=0.15  ***(4) Child psychomotor development***  Exposed 90.0 (11.4) Unexposed 98.2 (9.1), [5.55], P=0.02  Analysis of covariance | APGAR scores at 5 minutes |

*Between group differences were either reported in the paper or calculated from summary statistics provided (two-sided alpha=0.05 for t-test, chi-square), group listed (exposed, non-exposed) is the group with the statistically significant higher proportion / mean score for the listed characteristic, NGD indicates No Group Difference on the characteristics; some characteristics and between-group differences in characteristics were calculated using data reported in the paper

** as reported in the paper

BMI body mass index, aOR adjusted odds ratio; aHR adjusted hazard ratio, CI confidence interval, CV Cardiovascular, NSAID Nonsteroidal anti-inflammatory drugs, AD antidepressants, VAS visual analogue scale, NICU Neonatal Intensive Care Unit, APGAR each of the following; Activity, Pulse, Grimace, Appearance, Respiration assessed on a scale 0-2 X minutes after delivery and summed with higher APGAR scores reflecting better condition of the baby.

**Table S4. Quality assessment for neurodevelopmental and neurobehavioural outcomes**

|  | Selection /4 | Comparability /2 | | | | Outcome /2 | Total /8 |
| --- | --- | --- | --- | --- | --- | --- | --- |
|  |  | ½ point^a^ | ½ point^b^ | ½ point^c^ | ½ point^d^ |  |  |
| Suri (2011) | 4 | ½ | ½ | 0 | ½ | 2 | 7 ½ |
| El Marroun (2014) | 4 | 0 | ½ | ½ | ½ | 1 | 6 ½ |
| Pederson (2013) | 3 | 0 | ½ | ½ | ½ | 0 | 4 ½ |
| Nulman (2012) | 3 | 0 | 0 | 0 | 0 | 1 | 4 |
| Pederson (2010) | 3 | 0 | ½ | 0 | ½ | 1 | 5 |
| Santucci (2014) | 3 | 0 | 0 | 0 | 0 | 1 | 4 |
| Casper (2003) | 2 | 0 | 0 | 0 | 0 | 1 | 3 |

^a^ study controls for prenatal depression severity
^b^ study controls for depression severity at any point after delivery

^c^ study controls for socio-economic status (can be measured in income, deprivation score, education, home ownership etc. either pre- or postnatally)

^d^ study controls for any two of the following: (1) other psychoactive drug use during pregnancy, (2) smoking in pregnancy (3) drinking during pregnancy, (4) intrauterine growth restriction / preterm delivery / gestational age at delivery / SGA, (5) birth difficulties, (6) maternal age and sex of the child, (7) child second hand smoke exposure or other environmental pollution exposure, (8) child injury, (9) paternal/partner psychiatric disorder or symptoms, (10) further antidepressant exposure through breastfeeding, (11) breastfeeding, (12) maternal and/or paternal IQ.
