# Supplementary material for: A systematic review of maternal antidepressant use in pregnancy and short- and long-term offspring’s outcomes
Source: Arch Womens Ment Health. 2017 Oct 12;21(2):127–40. doi: 10.1007/s00737-017-0780-3 (PMC5856864; doi:10.1007/s00737-017-0780-3)
Supplement: Supplementary file 6 — (DOCX 21 kb) [file 737_2017_780_MOESM6_ESM.docx]

**Table S4. Quality assessment for neurodevelopmental and neurobehavioural outcomes**

|  | Selection /4 | Comparability /2 | | | | Outcome /2 | Total /8 |
| --- | --- | --- | --- | --- | --- | --- | --- |
|  |  | ½ point^a^ | ½ point^b^ | ½ point^c^ | ½ point^d^ |  |  |
| Suri (2011) | 4 | ½ | ½ | 0 | ½ | 2 | 7 ½ |
| El Marroun (2014) | 4 | 0 | ½ | ½ | ½ | 1 | 6 ½ |
| Pederson (2013) | 3 | 0 | ½ | ½ | ½ | 0 | 4 ½ |
| Nulman (2012) | 3 | 0 | 0 | 0 | 0 | 1 | 4 |
| Pederson (2010) | 3 | 0 | ½ | 0 | ½ | 1 | 5 |
| Santucci (2014) | 3 | 0 | 0 | 0 | 0 | 1 | 4 |
| Casper (2003) | 2 | 0 | 0 | 0 | 0 | 1 | 3 |

^a^ study controls for prenatal depression severity
^b^ study controls for depression severity at any point after delivery

^c^ study controls for socio-economic status (can be measured in income, deprivation score, education, home ownership etc. either pre- or postnatally)

^d^ study controls for any two of the following: (1) other psychoactive drug use during pregnancy, (2) smoking in pregnancy (3) drinking during pregnancy, (4) intrauterine growth restriction / preterm delivery / gestational age at delivery / SGA, (5) birth difficulties, (6) maternal age and sex of the child, (7) child second hand smoke exposure or other environmental pollution exposure, (8) child injury, (9) paternal/partner psychiatric disorder or symptoms, (10) further antidepressant exposure through breastfeeding, (11) breastfeeding, (12) maternal and/or paternal IQ.
